# Real-world antipsychotic prescribing pathways and treatment modifications in schizophrenia spectrum disorders: evidence from the United Arab Emirates

**DOI:** 10.3389/fphar.2026.1808636

**Published:** 2026-05-29

**Authors:** Syed Ali Bokhari, Muhanad Elnoor, Syed Fahad Javaid, Mohamed A. Alnor

**Affiliations:** 1 Al Amal Psychiatric Hospital, Emirates Health Services, Dubai, United Arab Emirates; 2 Department of Psychiatry, College of Medicine and Health Sciences, United Arab Emirates University, Al Ain, United Arab Emirates; 3 Department of Psychiatry, Faculty of Medicine, University of Khartoum, Khartoum, Sudan; 4 Department of Psychiatry, Al Ain Hospital, SEHA, Al Ain, United Arab Emirates

**Keywords:** antipsychotic prescribing, clozapine, long-acting injectable, polypharmacy, schizophrenia spectrum disorders, treatment persistence, United Arab Emirates

## Abstract

**Background:**

Antipsychotic prescribing patterns in Gulf Cooperation Council countries remain poorly characterised despite distinctive challenges including workforce shortages, high expatriate populations, and cultural factors influencing treatment engagement. This study characterised antipsychotic prescribing pathways in the largest Gulf-region cohort to date.

**Methods:**

This retrospective cohort study analysed electronic medical records of 5,125 patients with schizophrenia spectrum disorders (ICD-10 F20–F29) treated at Al Amal Psychiatric Hospital, Dubai, United Arab Emirates, between January 2018 and December 2025. Six domains were examined: first-line antipsychotic selection, treatment persistence, treatment modifications, long-acting injectable(s) (LAI) utilisation, clozapine prescribing, and antipsychotic polypharmacy. Kaplan–Meier survival analysis, Cox proportional hazards models, and logistic regression were employed, with propensity-score weighting for class comparisons.

**Results:**

The cohort (62.7% male; mean age 33.3 years) represented 120 nationalities. First-generation antipsychotics comprised 43.8% of all first-line prescriptions (predominantly intramuscular haloperidol), but second-generation antipsychotics accounted for 90.4% of first-line oral prescriptions, with olanzapine (43.5%) and risperidone (25.3%) predominating. Median persistence on the first oral antipsychotic was 36 days; only 23.0% remained at 12 months. Propensity-score weighted analysis confirmed a persistence disadvantage for oral first-generation antipsychotics (HR 1.43, 95% CI 1.21–1.70), largely attributable to haloperidol bridging therapy. Treatment modifications occurred in 60.1% of patients, with a median of 3 distinct antipsychotics (IQR 2–4), rising to 4 (IQR 3–5) in treatment-resistant patients. LAI utilisation was 34.1%, driven by treatment-resistant cases (55.3% vs 20.3%). Among 2,028 patients meeting treatment-resistant schizophrenia criteria (39.6%), only 7.8% received clozapine, with a median delay of 10.2 months from eligibility and 26.7% initiated within 3 months. Oral polypharmacy affected 42.9% of patients; cross-sectional point prevalence (20%–22%) was below global benchmarks. Benzodiazepine co-prescribing (79.8%) exceeded most international comparators, partly reflecting the acute inpatient setting and longitudinal measurement. Ethnic variation was observed across prescribing domains, and LAI initiation accelerated 19-fold in 2024–2025 *versus* 2018–2020.

**Conclusion:**

While oral first-line prescribing aligns with international guidelines, the findings highlight poor treatment persistence, substantial clozapine underutilisation, high longitudinal polypharmacy despite cross-sectional rates below international benchmarks, and ethnic variation across one of the world’s most diverse populations, identifying specific targets for quality improvement in Gulf psychiatric settings.

## 1 Introduction

Schizophrenia spectrum disorders affect approximately 24 million people worldwide and represent one of the leading causes of disability globally (Global Burden of Disease Collaborative Network, 2024). Antipsychotic medications remain the cornerstone of pharmacological treatment, yet substantial variation exists in prescribing patterns, treatment persistence, and clinical outcomes across healthcare systems (Leucht et al., 2013). Understanding these patterns is essential for identifying quality improvement priorities and informing evidence-based service planning. The evolution of antipsychotic pharmacotherapy over the past 3 decades has been characterised by a pronounced shift from first-generation antipsychotics (FGAs) to second-generation antipsychotics (SGAs), with global surveys documenting absolute increases of up to 50% in SGA utilisation (Hálfdánarson et al., 2017). However, the optimal balance between antipsychotic classes, appropriate use of long-acting injectable(s) (LAI) formulations, timely clozapine initiation for treatment-resistant cases, and management of antipsychotic polypharmacy remain areas of ongoing clinical and research interest (Correll et al., 2018; Correll et al., 2022).

International prescribing data reveal marked variation across each of these domains. SGA utilisation ranges from 75% to 93% in developed healthcare systems, though major clinical guidelines do not mandate either antipsychotic class as first-line treatment, and network meta-analyses demonstrate comparable efficacy between several FGAs and SGAs (American Psychiatric Association, 2020; Huhn et al., 2019). Treatment persistence is universally challenging: the Clinical Antipsychotic Trials of Intervention Effectiveness (CATIE) study demonstrated 74% discontinuation over 18 months (Lieberman et al., 2005), and registry-based studies report median time to discontinuation of 3.6–11.4 months depending on population and methodology (Tiihonen et al., 2017; Zacker et al., 2024). LAI utilisation varies from near-zero in some Asian countries to 68% in Singapore, with guidelines increasingly recommending earlier initiation (Chong et al., 2004; Correll et al., 2016). Clozapine, the most effective treatment for treatment-resistant schizophrenia (TRS), remains globally underutilised, with population-adjusted prescribing rates varying more than 300-fold between countries despite approximately 23%–34% of patients meeting TRS criteria (Bachmann et al., 2017; Siskind et al., 2022). Global antipsychotic polypharmacy prevalence is estimated at 33.2% for schizophrenia spectrum disorders, with substantial regional variation from 15% in North America to 39% in Africa (Højlund et al., 2024).

The Middle East and North Africa (MENA) region faces distinctive challenges in psychiatric service delivery that may further influence these prescribing patterns. Gulf Cooperation Council (GCC) countries have fewer than 3 psychiatrists per 100,000 population compared with the OECD average of 17.5 (OECD and World Health Organization, 2024; PwC, 2022), and an estimated 80% of mental health conditions in the region remain undiagnosed (Sharma et al., 2025). Cultural factors including stigma, beliefs regarding supernatural causation, and family decision-making dynamics influence help-seeking behaviour and treatment engagement (Zo et al., 2018).

The United Arab Emirates presents a particularly complex clinical context, with an approximately 88% expatriate population drawn from over 200 nationalities, creating one of the most ethnically diverse healthcare environments worldwide (Abbas Zaher et al., 2021; UAE Ministry of Foreign Affairs, 2025). Despite these unique characteristics, published pharmacoepidemiological data from the region remain sparse. The multi-country RECONNECT-S study (N = 1,057) and single-centre reports from Qatar and Oman represent the only available regional data, and none examined comprehensive treatment pathways, long-term prescribing outcomes, or ethnic variation in antipsychotic prescribing within a single healthcare system (Alkhadhari et al., 2015).

This study aimed to characterise antipsychotic prescribing patterns comprehensively in the largest Gulf-region cohort to date, comprising 5,125 patients with schizophrenia spectrum disorders representing 120 nationalities treated at a UAE tertiary psychiatric hospital over a 8-year period from 2018 to 2025. We examined six interrelated domains: first-line antipsychotic selection, treatment persistence and discontinuation, treatment modifications, long-acting injectable utilisation, clozapine prescribing, and antipsychotic polypharmacy. By benchmarking these patterns against international data from registries, clinical trials, and cross-national surveys, this study addresses a critical gap in regional psychiatric pharmacoepidemiology and provides the foundation for targeted quality improvement in antipsychotic prescribing across diverse Gulf healthcare settings.

## 2 Materials and methods

### 2.1 Study design and setting

This retrospective cohort study used electronic medical records (EMR) from Al Amal Psychiatric Hospital, the principal tertiary government psychiatric facility serving psychiatric referrals from Dubai and the five Northern Emirates (Sharjah, Ajman, Ras Al Khaimah, Umm Al Quwain, and Fujairah). The hospital’s catchment therefore encompasses six of the seven emirates of the United Arab Emirates and receives referrals from the community, general hospitals, and emergency departments across this area. Al Amal operates inpatient, outpatient, emergency and community psychiatric services, and is the endpoint of the specialist referral pathway for the catchment population. The study period extended from 1 January 2018 to 22 December 2025.

The study is reported in accordance with the Strengthening the Reporting of Observational Studies in Epidemiology (STROBE) statement for cohort studies (Von Elm et al., 2007). The completed STROBE checklist is provided as Supplementary Appendix.

### 2.2 Study population

Adult patients (≥18 years) were eligible if they had at least one recorded schizophrenia spectrum diagnosis (ICD-10 F20–F29) documented at any clinical encounter (including outpatient visits, emergency presentations, and inpatient admissions) and at least one antipsychotic prescription during the study period. After excluding 160 patients without prescription records, the final cohort comprised 5,125 patients. Analyses focusing on oral first-line treatment were restricted to patients whose first recorded antipsychotic was an oral formulation (N = 5,066).

Median follow-up duration was 2.3 years (IQR 0.8–4.7 years). Treatment era was evenly distributed: 34.2% of patients initiated treatment in 2018–2020, 46.5% in 2021–2023, and 19.3% in 2024–2025.

Missing data were minimal for core variables: age and sex were complete for all patients; nationality was missing for <1% and coded as 'Unknown'. Diagnostic codes were derived from clinician-assigned ICD-10 codes recorded in the electronic medical record.

### 2.3 Data Sources

Demographic, diagnostic, prescription, and encounter data were obtained from Al Amal Psychiatric Hospital’s Oracle Cerner Millennium EMR system, the hospital’s clinical documentation platform throughout the study period. The extract contained structured fields for patient demographics (age at encounter, sex, nationality), clinician-assigned ICD-10 diagnostic codes, prescription records (drug name, route of administration, dose, start and stop dates, prescribing encounter), and encounter metadata (visit type, encounter date). Diagnostic coding and prescribing were carried out as part of routine clinical care, entered directly by treating clinicians at the point of care.

A de-identified research extract covering all patients with any ICD-10 F20–F29 diagnosis during the study period was generated by senior clinical data analysts using structured query language (SQL) queries against the Cerner Millennium database. The analysts were external to the study team, were not involved in study design or interpretation, and were blinded to the study hypotheses. Direct patient identifiers were removed before release to the study team, in accordance with UAE data protection requirements and the conditions of the ethics approval. The retained extract was at the individual patient level, comprising a pseudonymised patient identifier together with demographic variables (age, sex, nationality) to permit within-patient linkage across encounters.

Before analysis, systematic data-quality checks were performed on the prescribing dataset, including verification of chronological consistency of prescription dates, duplicate-record screening, and confirmation of route classification for key antipsychotic exposures. Multiple EMR entries referring to the same antipsychotic agent (differing by strength or formulation descriptor) were mapped to a single canonical medication identifier through a structured procedure applied programmatically to the full dataset. Each prescription was classified by route of administration (oral, acute intramuscular referring to short-acting as-needed (pro re nata, PRN) injections for agitation, and long-acting injectable referring to depot maintenance formulations) and by antipsychotic class (first-generation *versus* second-generation antipsychotic). Long-acting injectable formulations were identified using product-level attributes (decanoate, palmitate, extended-release, monthly, three-monthly) independent of the prescribing encounter type, to avoid miscoding acute intramuscular doses as depot maintenance therapy.

Several features of the data generation and extraction process limit the risk of information bias. Prescribing and diagnostic data were generated as part of routine clinical workflow rather than for study-specific purposes, and therefore predate any analytic question. The data extract was prepared by analysts blinded to study aims, reducing the risk of selective extraction. Medication standardisation and route classification were applied programmatically to the full dataset rather than case-by-case, reducing adjudication bias. The analytic plan, including cohort definition, operational outcome definitions, multivariable covariate set, and sensitivity analyses, was pre-determined and documented before inspection of analytic results.

### 2.4 Outcomes and operational definitions

First-line antipsychotic selection was defined as the first antipsychotic recorded within the EMR system for each patient and was summarised both across all routes and for oral formulations only, to better approximate intended maintenance prescribing.

Treatment persistence on the first oral antipsychotic was defined as continuous exposure until discontinuation, with discontinuation defined as a gap of at least 60 days without a subsequent prescription. Patients with a single prescription and no refill were assigned a persistence of 0 days. Persistence was estimated using two complementary methods: the Fraction Continuous (FC) method calculated the proportion of patients remaining on treatment at each timepoint among those with sufficient follow-up to be evaluable; the Kaplan-Meier (KM) method provided censoring-adjusted survival estimates that account for patients with incomplete follow-up. This operational definition captures continuity of recorded prescribing within the EMR and should not be interpreted as direct confirmation of medication adherence or ingestion.

Treatment modification was assessed as switching and/or augmentation. Switching referred to changing from one antipsychotic to another, whereas augmentation referred to adding a further antipsychotic to the existing regimen. Modifications were summarised by the number of switches and the maximum treatment step reached.

Long-acting injectable(s) (LAI) utilisation was defined as receipt of any LAI antipsychotic during follow-up; the specific agent(s) used and time from first oral antipsychotic to first LAI were recorded.

Clozapine initiation was identified from prescription records and time to clozapine was measured from the first recorded antipsychotic prescription to clozapine start. Patients whose first recorded antipsychotic was clozapine were described separately as likely transfers with prior undocumented treatment.

Antipsychotic polypharmacy was defined as receipt of two or more distinct antipsychotics prescribed within a 30-day window. Polypharmacy was evaluated both across all routes and for oral prescriptions only.

Psychiatric comorbidities were identified using ICD-10 diagnostic codes recorded at or before the date of first antipsychotic prescription. Substance use disorder (SUD) was defined as any F10–F19 diagnosis. Other psychiatric comorbidities assessed included anxiety disorders (F40–F41), depressive disorders (F32–F33), and personality disorders (F60–F69). Comorbidities accrued after treatment initiation were not included in baseline characterisation.

### 2.5 Treatment-resistant schizophrenia definition

Treatment-resistant schizophrenia (TRS) was operationally defined from EMR prescribing data as failure to respond to at least two adequate antipsychotic trials, consistent with international consensus criteria (Global Burden of Disease Collaborative Network, 2024). An adequate trial was defined as treatment with an antipsychotic at a therapeutic dose (defined as the midpoint or above of the licensed dose range, or chlorpromazine equivalent ≥400 mg/day) for at least ≥28 days (4 weeks) (Howes et al., 2017; Remington et al., 2017; John et al., 2018). This threshold was selected to reflect real-world prescribing and switching patterns in routine care and is consistent with accepted adequacy standards that commonly cite 4–6 weeks as a minimum trial duration. Because symptom response, tolerability, and adherence could not be directly adjudicated from prescribing records alone, this definition should be understood as a pragmatic electronic medical record proxy for probable treatment resistance rather than a clinically validated diagnosis.

Although some consensus guidelines specify a minimum duration of 6 weeks for an adequate antipsychotic trial (Howes et al., 2017; Kane et al., 2019), a 4-week threshold was selected to reflect real-world clinical practice and pharmacoepidemiological conventions, where medication changes frequently occur earlier due to clear non-response, intolerable adverse effects, or clinical deterioration (Remington et al., 2017). A stricter sensitivity definition requiring failure of at least three adequate trials was also applied (Howes et al., 2017; Kane et al., 2019).

### 2.6 Statistical analysis

Continuous variables are presented as mean (SD) or median (IQR), and categorical variables as n (%). Group comparisons used chi-square or Fisher’s exact tests for categorical variables and Mann–Whitney U tests for continuous variables. Time-to-event outcomes (persistence, time to switching, time to LAI initiation, and time to clozapine) were analysed using Kaplan–Meier methods with log-rank tests and multivariable Cox proportional hazards models adjusted for demographic and clinical covariates. Logistic regression was used for binary outcomes (any LAI receipt; any clozapine receipt) with similar covariate adjustment.

To compare persistence between first-line oral first-generation versus second-generation antipsychotics, inverse probability of treatment weighting was applied using a propensity score model including age, sex, nationality (UAE national versus expatriate), primary diagnosis, treatment era, and psychiatric comorbidities. Covariate balance was assessed using standardised mean differences and weighted Cox regression was used to estimate the hazard ratio for discontinuation.

Analyses were performed using Python (version 3.13) with the following packages: pandas (2.2.3) for data management, lifelines (0.30.0) for Kaplan-Meier survival analysis and Cox proportional hazards models, statsmodels (0.14.4) for logistic regression, scipy (1.15.3) for statistical tests, and scikit-learn (1.6.1) for propensity score estimation. Proportional hazards assumptions for Cox models were assessed using residual-based diagnostics. Collinearity among regression covariates was evaluated using variance inflation factors. The index date for all time-to-event analyses was the date of first recorded antipsychotic prescription within the EMR system. Complete-case analysis was used for all multivariable models. Statistical significance was set at two-sided p < 0.05; secondary and subgroup analyses should be considered exploratory.

### 2.7 Sensitivity analyses

Sensitivity analyses examined: (1) alternative discontinuation-gap definitions (30, 60, and 90 days); (2) restriction to patients with at least 12 months of follow-up; (3) exclusion of patients with fewer than three prescriptions or fewer than two clinical encounters, to assess whether single-encounter or transient patients biased persistence estimates; (4) restriction by diagnostic subgroup, including schizophrenia (F20) only and excluding brief psychotic disorder (F23); (5) stricter TRS criteria using a six-week minimum trial duration; (6) exclusion of patients entering care after October 2025, after July 2025, and before 2024 to examine whether limited observation time among recent entrants materially affected persistence estimates; and (7) propensity-score weighted comparisons with and without oral haloperidol to evaluate whether the FGA persistence disadvantage reflected bridging therapy rather than a true class effect.

### 2.8 Ethical considerations

Ethical approval for this retrospective study was obtained from the Ministry of Health and Prevention Research Ethics Committee, United Arab Emirates (MOHAP/DXB-REC/M.J.J/No. 91/2024). The requirement for informed consent was waived because the study involved secondary analysis of routinely collected clinical data and posed minimal risk to participants. All data were de-identified prior to analysis, and the study was conducted in accordance with applicable institutional and national ethical standards and the principles of the Declaration of Helsinki.

## 3 Results

### 3.1 Study population

**FIGURE 1:**
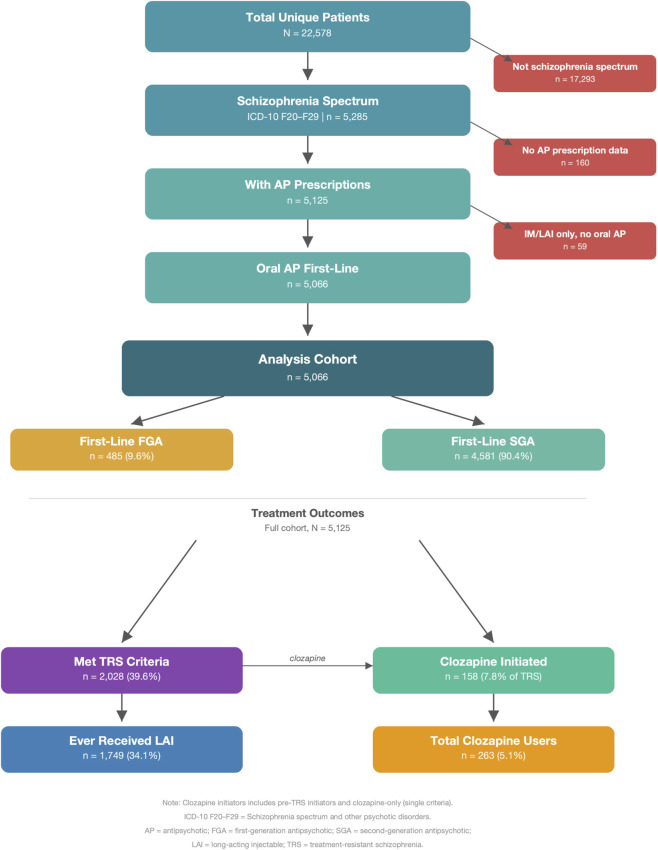
Patient selection flow diagram. Note. Derivation of the study cohort from the psychiatric database. Upper boxes show the number of patients at each selection step with reasons for exclusion. The final cohort included 5,125 patients with schizophrenia spectrum diagnoses who had any antipsychotic exposure. Lower panels summarise key treatment outcomes: treatment-resistant schizophrenia (n = 2,028; 39.6%), clozapine initiation among treatment-resistant schizophrenia patients (n = 158; 7.8%), long-acting injectable antipsychotic receipt (n = 1,749; 34.1%), and total clozapine users (n = 263; 5.1%, including pre-treatment-resistant schizophrenia and single-criterion initiators). AP, antipsychotic; ICD-10, International Classification of Diseases, Tenth Revision; LAI, long-acting injectable; TRS, treatment-resistant schizophrenia.

Of 22,578 unique patients in the psychiatric database, 5,285 (23.4%) had schizophrenia spectrum diagnoses (ICD-10 F20–F29). After excluding 160 patients without antipsychotic prescription data, 5,125 patients had any antipsychotic exposure and comprised the primary analysis cohort (Figure 1). Of these, 5,066 patients (98.8%) had oral antipsychotic prescription data and were included in the oral-specific analyses; the remaining 59 patients received only intramuscular or long-acting injectable formulations as first treatment. The cohort was 62.7% male, with a mean age of 33.3 years (SD 9.6; median 32.0, IQR 26.0–39.0).

The population represented 120 nationalities across six continents. The 15 most common nationalities are shown in Table 1, with Emirati nationals comprising 26.2% (n = 1,327), followed by Indian (14.9%, n = 757), Pakistani (7.0%, n = 357), Ethiopian (4.9%, n = 246), and Egyptian (3.7%, n = 185) patients.

**TABLE 1:** Baseline demographic and clinical characteristics of the oral first-line study cohort.

Characteristic	Overall (N = 5,066)	FGA (N = 485)	SGA (N = 4,581)	P-value	SMD
Demographics					
Age (years) (mean ± SD)	33.3 ± 9.6	33.4 ± 9.8	33.3 ± 9.5	0.598	0.013
Sex	​	​	​	0.708	0.012
Male	3,181 (62.8%)	302 (62.3%)	2,879 (62.8%)	​	​
Female	1,885 (37.2%)	183 (37.7%)	1,702 (37.2%)	​	​
Nationality					
Emirati	1,327 (26.2%)	116 (23.9%)	1,211 (26.4%)	**0.019**	0.058
Indian	757 (14.9%)	56 (11.5%)	701 (15.3%)		0.110
Pakistani	357 (7.0%)	27 (5.6%)	330 (7.2%)		0.067
Ethiopian	246 (4.9%)	32 (6.6%)	214 (4.7%)		0.084
Egyptian	185 (3.7%)	31 (6.4%)	154 (3.4%)		0.141
Sudanese	179 (3.5%)	19 (3.9%)	160 (3.5%)		0.022
Bangladeshi	161 (3.2%)	20 (4.1%)	141 (3.1%)		0.056
Ugandan	129 (2.5%)	17 (3.5%)	112 (2.4%)		0.062
Syrian	114 (2.3%)	8 (1.6%)	106 (2.3%)		0.048
Filipino	99 (2.0%)	9 (1.9%)	90 (2.0%)		0.008
Jordanian	94 (1.9%)	7 (1.4%)	87 (1.9%)		0.036
Yemeni	93 (1.8%)	11 (2.3%)	82 (1.8%)		0.034
Nepalese	85 (1.7%)	7 (1.4%)	78 (1.7%)		0.021
Nigerian	73 (1.4%)	10 (2.1%)	63 (1.4%)		0.053
Comoran	71 (1.4%)	4 (0.8%)	67 (1.5%)		0.060
Other	1,096 (21.6%)	111 (22.9%)	985 (21.5%)		0.033
Era of first antipsychotic					
2018–2020	1,733 (34.2%)	71 (14.6%)	1,662 (36.3%)	**<0.001**	0.513
2021–2023	2,356 (46.5%)	149 (30.7%)	2,207 (48.2%)		0.363
2024–2025	977 (19.3%)	265 (54.6%)	712 (15.5%)		0.898
Clinical Characteristics					
Primary Diagnosis					
Schizophrenia	2,301 (45.4%)	215 (44.3%)	2,086 (45.5%)	**<0.001**	0.024
Brief Psychotic Disorder	1,806 (35.6%)	180 (37.1%)	1,626 (35.5%)		0.034
Other Psychotic Disorder	450 (8.9%)	40 (8.2%)	410 (9.0%)		0.025
Schizoaffective	342 (6.8%)	35 (7.2%)	307 (6.7%)		0.020
Delusional Disorder	165 (3.3%)	15 (3.1%)	150 (3.3%)		0.010
Other Schizophrenia Spectrum	2 (0.0%)	0 (0.0%)	2 (0.0%)		0.000
Meets TRS criteria, n (%)	2,028 (40.0%)	242 (49.9%)	1,786 (39.0%)	**<0.001**	0.221
Comorbid SUD, n (%)	766 (15.1%)	85 (17.5%)	681 (14.9%)	0.116	0.072
Medications					
Number of distinct APs (mean ± SD)	3.0 ± 1.7	3.1 ± 1.5	3.0 ± 1.7	**0.038**	0.041
Number of distinct APs (median, IQR)	3.0 (2.0–4.0)	3.0 (2.0–4.0)	3.0 (2.0–4.0)	​	​
Concomitant Medications					
Mood stabilizer use	1,370 (27.0%)	129 (26.6%)	1,241 (27.1%)	0.832	0.011
Antidepressant use	1,656 (32.7%)	133 (27.4%)	1,523 (33.2%)	**0.003**	0.127
Benzodiazepine use	4,054 (80.0%)	436 (89.9%)	3,618 (79.0%)	**<0.001**	0.305
Anticholinergic use	3,391 (66.9%)	436 (89.9%)	2,955 (64.5%)	**<0.001**	0.635

**Bold** p-values indicate statistical significance (p < 0.05). Data are presented as n (%) or median (IQR) unless otherwise specified. P-values were calculated using chi-square tests for categorical variables and Mann-Whitney U tests for continuous variables. SMD, standardised mean difference, calculated to assess balance between FGA- and SGA-initiated groups; values > 0.1 indicate meaningful imbalance. Abbreviations: FGA, first-generation antipsychotic; SGA, second-generation antipsychotic; TRS, treatment-resistant schizophrenia; SUD, substance use disorder; SD, standard deviation; IQR, interquartile range.

Diagnostic categorisation showed that 46.1% (n = 2,364) had a schizophrenia diagnosis in their clinical history and 15.0% (n = 770) had a schizoaffective disorder diagnosis. The remaining patients had other schizophrenia spectrum diagnoses including brief psychotic disorder, delusional disorder, and other psychotic disorders. TRS, defined as failure of two or more adequate antipsychotic trials, was identified in 39.6% of the cohort (n = 2,028) using the 4-week adequate trial definition, and 31.6% (n = 1,617) using the 6-week definition.

Comorbid substance use disorder (SUD) was documented in 15.1% of patients (n = 766), with numerically higher rates among FGA-initiated patients (17.7%) compared to SGA-initiated patients (14.9%), though this difference did not reach statistical significance (p = 0.116). Concomitant psychotropic medication use was extensive across the full cohort (N = 5,125): benzodiazepines were prescribed to 79.8% of patients (n = 4,088), anticholinergics to 66.7% (n = 3,417), antidepressants to 32.5% (n = 1,666), and mood stabilisers to 26.8% (n = 1,376). Both benzodiazepine use (89.9% vs 79.0%, p < 0.001) and anticholinergic use (90.1% vs 64.5%, p < 0.001) were higher among first-generation antipsychotic-initiated patients.

### 3.2 First-line antipsychotic selection

Considering all routes of administration, first-generation antipsychotics (FGAs) were prescribed as first-line treatment in 2,246 patients (43.8%), while second-generation antipsychotics (SGAs) were first-line in 2,879 patients (56.2%) (Table 2).

**TABLE 2:** First-line antipsychotic prescribing by route (N = 5,125).

A. First-line antipsychotic (all routes)				
Antipsychotic	N	%	Class	LAI
Haloperidol	2,205	43.0%	FGA	No
Olanzapine	1,332	26.0%	SGA	No
Risperidone	664	13.0%	SGA	No
Aripiprazole	435	8.5%	SGA	No
Quetiapine	271	5.3%	SGA	No
Paliperidone LAI	73	1.4%	SGA	Yes
Clozapine	69	1.3%	SGA	No
Ziprasidone	20	0.4%	SGA	No
Trifluoperazine	15	0.3%	FGA	No
Flupenthixol	14	0.3%	FGA	No
Lurasidone	9	0.2%	SGA	No
Chlorpromazine	8	0.2%	FGA	No
Aripiprazole LAI	4	0.1%	SGA	Yes
Promazine	4	0.1%	FGA	No
Paliperidone	2	0.0%	SGA	No

B. First oral antipsychotic				
Antipsychotic	N	%	Generation	
Olanzapine	2,204	43.5%	SGA	
Risperidone	1,283	25.3%	SGA	
Aripiprazole	628	12.4%	SGA	
Haloperidol	450	8.9%	FGA	
Quetiapine	358	7.1%	SGA	
Clozapine	71	1.4%	SGA	
Ziprasidone	24	0.5%	SGA	
Trifluoperazine	16	0.3%	FGA	
Chlorpromazine	10	0.2%	FGA	
Lurasidone	10	0.2%	SGA	
Promazine	5	0.1%	FGA	
Flupenthixol	4	0.1%	FGA	
Paliperidone	3	0.1%	SGA	
**Total Oral First-Line**	**5,066**	**100.0%**	​	
**SGA oral first-line**	**4,581**	**90.4%**	​	
**FGA oral first-line**	**485**	**9.6%**	​	

**(A)** Shows the first antipsychotic recorded for each patient across all routes of administration (oral, intramuscular, and long-acting injectable). The high proportion of haloperidol largely reflects intramuscular use for acute agitation management rather than intended maintenance therapy. **(B)** Shows the distribution of the first oral antipsychotic among patients whose initial treatment included an oral formulation (N = 5,066); 59 patients whose first recorded antipsychotic was intramuscular-only or long-acting injectable-only were excluded. Data are presented as n (%). Abbreviations: FGA, first-generation antipsychotic; SGA, second-generation antipsychotic; LAI, long-acting injectable(s).

Haloperidol was the single most commonly prescribed first-line antipsychotic when considering all routes (43.0%, n = 2,205), followed by olanzapine (26.0%, n = 1,332), risperidone (13.0%, n = 664), aripiprazole (8.5%, n = 435), and quetiapine (5.3%, n = 271). The majority of haloperidol prescriptions were intramuscular formulations.

**FIGURE 2:**
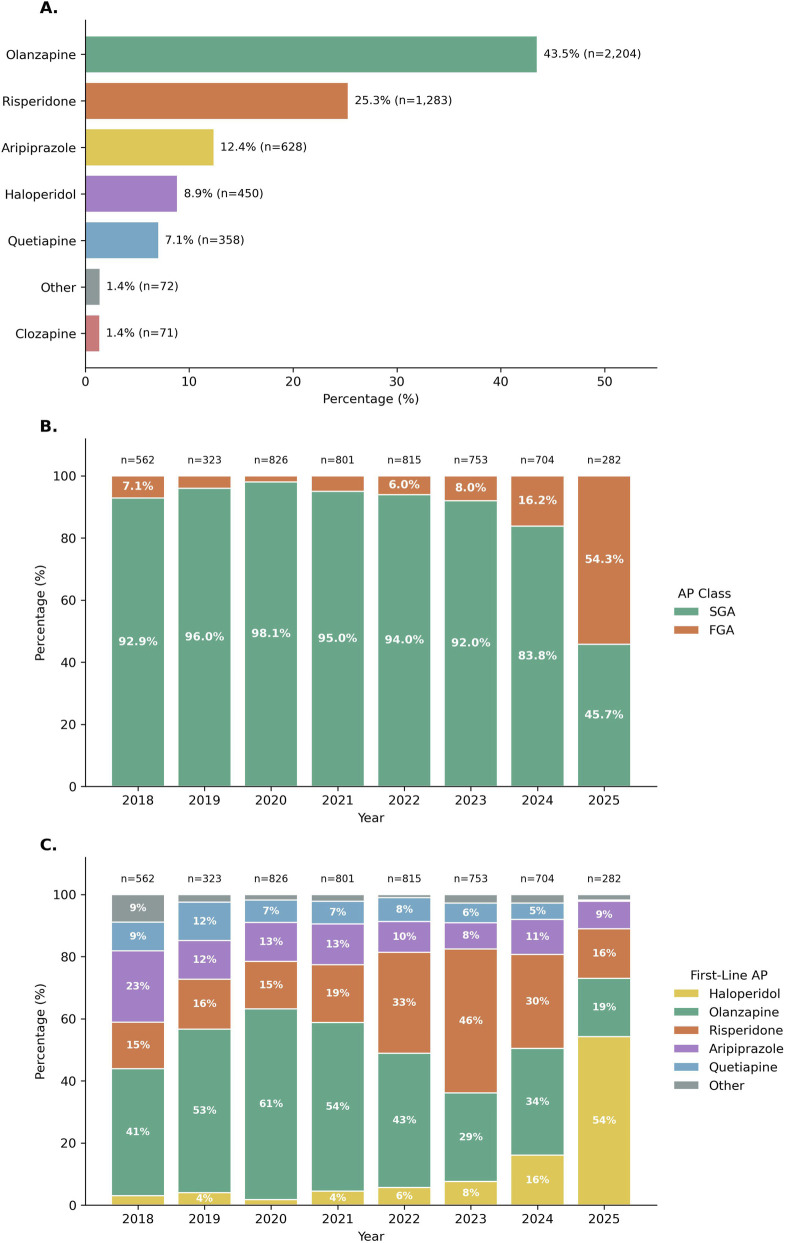
First-line antipsychotic prescribing patterns (N = 5,066). Note. Distribution and temporal trends in first-line oral antipsychotic selection among 5,066 patients with schizophrenia spectrum disorders. **(A)** Overall distribution of first-line oral antipsychotics. **(B)** Annual oral first-generation antipsychotic versus second-generation antipsychotic selection, showing a shift toward first-generation antipsychotic-first prescribing from 7.1% in 2018 to 54.3% in 2025. **(C)** Individual agent trends by year. Sample sizes are shown above bars. AP, antipsychotic; FGA, first-generation antipsychotic; SGA, second-generation antipsychotic.

A secondary analysis restricted to first oral antipsychotic prescriptions showed a different distribution (Figure 2A). Olanzapine was the most common first-line oral agent at 43.5% (n = 2,204), followed by risperidone (25.3%, n = 1,283), aripiprazole (12.4%, n = 628), haloperidol (8.9%, n = 450), and quetiapine (7.1%, n = 358). SGAs accounted for 90.4% of first-line oral prescriptions.

Variation in first-line prescribing was observed across ethnic groups. Olanzapine predominated among African patients (Ethiopian 62.5%, Ugandan 58.9%, Sudanese 55.0%) and South Asian patients (Indian 55.2%, Pakistani 54.7%, Bangladeshi 52.2%). In contrast, Emirati patients showed more balanced prescribing with higher aripiprazole (15.2%) and quetiapine (12.4%) use.

Diagnostic differences were apparent in first-line prescribing. Patients with schizophrenia were more likely to receive SGAs first-line (64.2%) compared to those with schizoaffective disorder (61.3%).

### 3.3 Temporal trends

**FIGURE 3:**
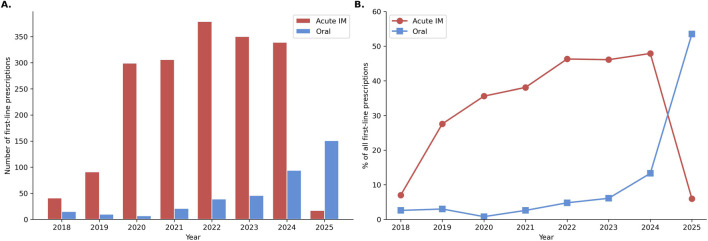
First-line prescribing route trends by calendar year. Note. Temporal trends in first-line antipsychotic prescribing route from 2018 to 2025. **(A)** Annual number of first-line prescriptions recorded as acute intramuscular or oral antipsychotic prescriptions. **(B)** Annual percentage of all first-line prescriptions accounted for by acute intramuscular and oral formulations. Acute intramuscular prescribing increased between 2020 and 2024 and declined sharply in 2025, while oral prescribing increased and became dominant in 2025. IM, intramuscular.

Substantial changes in first-line prescribing patterns were observed over the study period. Considering all routes, SGA first-line use declined from 86.0% in 2018 to 40.4% in 2025, while FGA first-line use increased from 14.0% to 59.6%; this was predominantly driven by intramuscular haloperidol for acute agitation management between 2018 and 2024 (Supplementary Table S1), whereas 2025 showed a shift toward oral haloperidol prescribing (Figure 3). Within oral prescriptions specifically, SGA oral first-line use declined from 92.9% in 2018 to 45.7% in 2025, while FGA oral first-line use increased from 7.1% to 54.3%. Oral haloperidol increased from 3.0% to 54.3%, while olanzapine concurrently declined (40.9%–18.8%). Risperidone remained relatively stable (14.9%–16.0%).

Time-to-event analyses revealed significantly faster treatment escalation in recent cohorts. Cox regression for time to LAI initiation showed patients treated in 2024–2025 reached LAI 19-fold faster than those in 2018–2020 (HR 19.14, 95% CI 15.46–23.69, p < 0.001). Patients in the 2021–2023 era showed similarly accelerated LAI initiation (HR 7.06, 95% CI 6.00–8.32, p < 0.001).

### 3.4 Treatment persistence

**FIGURE 4:**
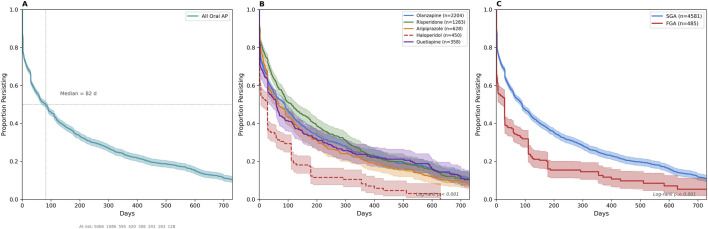
Kaplan-Meier survival curves for treatment persistence. Note. Time to discontinuation of first oral antipsychotic treatment. **(A)** Overall cohort persistence curve (N = 5,066) with 95% confidence interval shown as the shaded area; median persistence was 82 days by Kaplan-Meier estimate. Numbers at risk are shown below. **(B)** Persistence stratified by first-line antipsychotic agent (log-rank p < 0.001). Haloperidol showed the shortest persistence, whereas risperidone and quetiapine showed the longest median persistence. **(C)** Persistence by antipsychotic class (second-generation antipsychotic n = 4,581 versus first-generation antipsychotic n = 485; log-rank p < 0.001). Persistence was defined as continuous treatment without a gap exceeding 60 days. FGA, first-generation antipsychotic; SGA, second-generation antipsychotic.

Treatment persistence on the first oral antipsychotic was poor (Figure 4; Table 3). The raw median persistence was 36 days (IQR 6–86 days). However, because 38.8% of patients remained on their first antipsychotic at last follow-up (right-censored), Kaplan-Meier analysis estimated a censoring-adjusted median persistence of 82 days. The persistence curve showed steep early attrition. KM persistence was 70.7% at 14 days, dropping to 61.9% at 30 days, 53.3% at 60 days, 47.3% at 90 days, 35.1% at 6 months, and 23.0% at 12 months. The corresponding FC estimates (among patients with sufficient follow-up to be evaluable) were 68.3% at 30 days, 42.3% at 90 days, 27.7% at 6 months, and 16.6% at 12 months (Table 3).

**TABLE 3:** Treatment persistence on first oral antipsychotic.

First-line oral antipsychotic	N	FC 14d	KM 14d	FC 30d	KM 30d	FC 60d	KM 60d	FC 90d	KM 90d	FC 180d	KM 180d	FC 365d	KM 365d	FC median (IQR)	Mean ± SD	KM median
Overall	5,066	2643/3532 (74.8%)	70.7%	2090/3059 (68.3%)	61.9%	1337/2666 (50.2%)	53.3%	1060/2504 (42.3%)	47.3%	625/2260 (27.7%)	35.1%	328/1977 (16.6%)	23.0%	36 (6–86)	96.9 ± 196.8	82
Olanzapine	2,204	1048/1420 (73.8%)	69.0%	787/1165 (67.6%)	62.4%	473/970 (48.8%)	54.1%	385/915 (42.1%)	48.5%	216/828 (26.1%)	35.6%	112/725 (15.4%)	23.5%	35 (4–69)	83.6 ± 177.6	89
Risperidone	1,283	709/860 (82.4%)	78.2%	580/767 (75.6%)	71.6%	353/655 (53.9%)	60.7%	259/613 (42.3%)	53.1%	156/550 (28.4%)	41.0%	71/474 (15.0%)	24.8%	40 (15–83)	92.9 ± 169.2	111
Aripiprazole	628	396/508 (78.0%)	73.0%	324/480 (67.5%)	59.4%	227/450 (50.4%)	50.6%	195/431 (45.2%)	45.2%	109/395 (27.6%)	32.8%	61/361 (16.9%)	20.2%	36 (10–129)	123.2 ± 219.4	66
Haloperidol	450	167/314 (53.2%)	53.0%	104/238 (43.7%)	36.8%	61/191 (31.9%)	32.4%	43/162 (26.5%)	29.4%	15/129 (11.6%)	12.3%	7/95 (7.4%)	8.2%	20 (0–44)	43.7 ± 80.0	29
Quetiapine	358	214/308 (69.5%)	66.7%	196/293 (66.9%)	56.6%	145/285 (50.9%)	48.9%	112/277 (40.4%)	41.5%	80/264 (30.3%)	33.5%	47/239 (19.7%)	23.8%	38 (3–142)	144.5 ± 258.3	60
Clozapine	71	53/58 (91.4%)	88.7%	48/55 (87.3%)	75.4%	39/54 (72.2%)	66.2%	33/49 (67.3%)	62.9%	26/43 (60.5%)	58.9%	18/37 (48.6%)	41.2%	93 (30–348)	342.9 ± 538.2	296

Persistence defined as continuous treatment without a prescription gap exceeding 60 days. Two complementary methods are reported: the Fraction Continuous (FC) method calculates the proportion remaining on treatment among patients with sufficient follow-up at each timepoint (numerator/denominator shown in parentheses); the Kaplan-Meier (KM) method provides censoring-adjusted survival estimates accounting for patients with incomplete follow-up. FC, median represents the raw median persistence; KM, Median represents the censoring-adjusted median (time at which 50% discontinued). Abbreviations: IQR, interquartile range; SD, standard deviation.

Substantial differences in persistence were observed by antipsychotic class. Oral haloperidol showed lower persistence than SGAs: KM 14-day persistence was 53.0% for haloperidol compared to 69.0% for olanzapine, 78.2% for risperidone, and 73.0% for aripiprazole. At 12 months, 8.2% of haloperidol patients remained on treatment compared to 20.2%–24.8% for SGAs. Median persistence was 20 days for haloperidol *versus* 35–40 days for SGAs.

Among individual oral antipsychotics, risperidone showed the highest median persistence (40 days), followed by quetiapine (38 days), aripiprazole (36 days), and olanzapine (35 days). At 12 months, risperidone had the highest persistence (24.8%), followed by olanzapine (23.5%), quetiapine (23.8%), and aripiprazole (20.2%). Oral haloperidol had the lowest persistence (median 20 days, 12-month 8.2%). Persistence also varied by diagnosis (Table 3). Patients with schizophrenia showed median persistence of 45 days (IQR 9–119) compared to 38 days (IQR 4–100) for schizoaffective disorder and 30 days (IQR 3–38) for brief psychotic disorder.

### 3.5 Treatment modifications

Treatment modifications occurred in the majority of patients during follow-up (Table 4). Any treatment change occurred in 60.1% (n = 3,045) of patients with oral first-line treatment, with augmentation (52.0%, n = 2,635) more common than switching (18.7%, n = 947). The median time to first modification was 10 days (IQR 0–130).

**TABLE 4:** Treatment modifications during follow-up (N = 5,066).

Metric	Value
Any treatment change, n (%)	3,045 (60.1%)
Time to first change (days), median (IQR)	10 (0–130)
Switch (stopped first AP), n (%)	947 (18.7%)
Augmentation (added AP), n (%)	2,635 (52.0%)
FGA Oral First-Line (n = 485)	
Any change, n (%)	430 (88.7%)
Switch, n (%)	219 (46.1%)
Augmentation, n (%)	204 (42.9%)
SGA Oral First-Line (n = 4,581)	
Any change, n (%)	2,615 (57.1%)
Switch, n (%)	728 (15.9%)
Augmentation, n (%)	2,431 (53.0%)

Treatment modification defined as switching (discontinuation of initial antipsychotic and initiation of a different agent) or augmentation (addition of a second antipsychotic while continuing the first). Analysis restricted to patients with oral first-line antipsychotic. Time measured from first oral antipsychotic prescription. FGA, first-generation antipsychotic; SGA, second-generation antipsychotic; IQR, interquartile range.

Significant differences emerged between FGA-initiated and SGA-initiated patients. Those starting on oral FGAs had higher modification rates (88.7% vs 57.1%, p < 0.001) and were nearly three times more likely to switch agents entirely (46.1% vs 15.9%, p < 0.001).

Cox proportional hazards analysis identified predictors of time to first treatment modification (C-index 0.62; N = 5,066, events = 2,315). First-line haloperidol was the strongest predictor of earlier treatment modification (HR 3.08, 95% CI 2.69–3.53, p < 0.001), while first-line risperidone was associated with lower modification risk compared to olanzapine (HR 0.86, 95% CI 0.77–0.96, p = 0.008). First-line aripiprazole (HR 1.16, 95% CI 1.02–1.32, p = 0.022) and quetiapine (HR 1.26, 95% CI 1.09–1.46, p = 0.002) were associated with moderately higher modification rates compared to olanzapine. UAE nationality was associated with faster modification (HR 1.23, 95% CI 1.12–1.34, p < 0.001), as was treatment era 2021–2023 compared to 2018–2020 (HR 1.23, 95% CI 1.12–1.36, p < 0.001). Conversely, TRS status was associated with slower modification (HR 0.89, 95% CI 0.81–0.98, p = 0.015).

**FIGURE 5:**
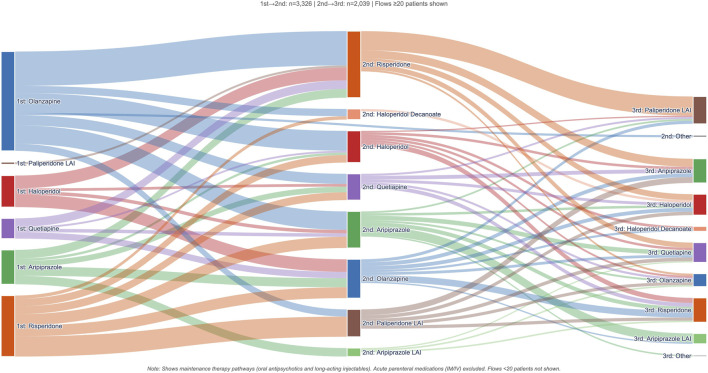
Antipsychotic treatment pathways. Note. Sankey diagram illustrating treatment transitions from first-line to second-line to third-line antipsychotic therapy among patients with treatment modifications (first-to-second transition: n = 3,326; second-to-third transition: n = 2,039). Shows maintenance therapy pathways including oral antipsychotics and long-acting injectable antipsychotic formulations. Acute parenteral medications, including intramuscular and intravenous medications, were excluded. Flow width is proportional to the number of patients. Flows with fewer than 20 patients are not shown. LAI, long-acting injectable(s). Transition counts represent pathway steps and may exceed the number of unique patients, as individual patients may contribute to multiple transitions.

The Sankey diagram (Figure 5) illustrates common treatment pathways for maintenance therapy (oral and LAI, excluding acute IM/IV). The predominant pattern was olanzapine to risperidone transition, accounting for 12.6% of all modifications. Risperidone to paliperidone LAI (7.4%) and olanzapine to haloperidol (7.3%) were also common.

Patients received a median of 3 distinct antipsychotics (IQR 2–4) during follow-up. The distribution was: 18.9% received one antipsychotic, 30.6% received two, 19.3% received three, 13.4% received four, and 17.7% received five or more distinct agents. TRS patients received significantly more antipsychotics (median 4, IQR 3–5) compared to non-TRS patients (median 2, IQR 1–3, p < 0.001).

### 3.6 Long-acting injectable utilisation

One-third of patients (n = 1,749, 34.1%) received a long-acting injectable(s) (LAI) antipsychotic at some point during follow-up (Table 5). By total prescription volume, paliperidone palmitate was the most commonly prescribed (59.8% of all LAI prescriptions, n = 20,204), followed by aripiprazole LAI (30.9%, n = 10,432) and haloperidol decanoate (9.3%, n = 3,157).

**TABLE 5:** Long-acting injectable antipsychotic utilisation.

Metric	Value
Ever received LAI	1,749 (34.1%)
Time to first LAI (days), median (IQR) - among non-first-line LAI	22.0 (5.0–200.0)
LAI formulation distribution (by prescription count)	
Paliperidone LAI	20,204 (59.8%)
Aripiprazole LAI	10,432 (30.9%)
Haloperidol Decanoate	3,157 (9.3%)
Flupenthixol Decanoate	18 (0.1%)
Zuclopenthixol Decanoate	2 (0.0%)

LAI, use presented as n (%) for patients or as total prescription counts for formulation distribution. Percentages for individual LAI, formulations are calculated based on total LAI prescriptions (N = 33,813), not number of patients. Time to LAI, initiation calculated from first oral antipsychotic, excluding patients who received LAI, as first treatment. Abbreviations: LAI, long-acting injectable(s); IQR, interquartile range.

Among patients who received LAI after their first oral antipsychotic (excluding LAI-first patients), median time to LAI initiation was 22 days (IQR 5–200 days).

LAI utilisation showed marked variation by clinical factors. Among TRS patients, 55.3% (n = 1,121) received LAI compared to 20.3% (n = 628) of non-TRS patients (p < 0.001). Patients with schizoaffective disorder had higher LAI rates (66.6%) compared to those with schizophrenia (44.7%).

**FIGURE 6:**
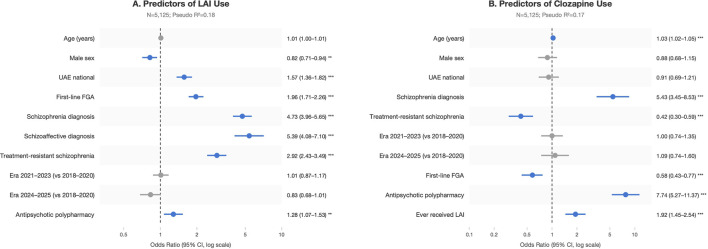
Multivariable independent predictors of long-acting injectable antipsychotic use and clozapine use. Note. Forest plots showing adjusted odds ratios (ORs) with 95% confidence intervals from multivariable logistic regression. **(A)** Multivariable logistic regression model for long-acting injectable antipsychotic predictors, excluding the number of distinct prior antipsychotics to resolve collinearity with treatment-resistant status. **(B)** Predictors of ever receiving clozapine. Reference line at OR = 1.0. Gray = non-significant (p ≥ 0.05); blue = significant (p < 0.05). Significance markers: **p < 0.01; ***p < 0.001. LAI, long-acting injectable(s).

Multivariable logistic regression identified several independent predictors of LAI initiation (Figure 6A; N = 5,125, Pseudo *R* ^2^ = 0.18). To avoid collinearity between treatment-resistant status and number of prior antipsychotics (which forms the basis of the TRS definition), the primary model excluded the prior antipsychotic count. In this model, TRS was a strong independent predictor of LAI initiation (OR 2.92, 95% CI 2.43–3.49, p < 0.001), as were schizophrenia diagnosis (OR 4.73, 95% CI 3.96–5.65, p < 0.001), schizoaffective disorder (OR 5.39, 95% CI 4.08–7.10, p < 0.001), and first-line FGA use (OR 1.96, 95% CI 1.71–2.26, p < 0.001). UAE nationality was associated with higher LAI receipt (OR 1.57, 95% CI 1.36–1.82, p < 0.001), while male sex was associated with lower LAI receipt (OR 0.82, 95% CI 0.71–0.94, p = 0.004). Treatment era and age were not significant independent predictors after adjustment.

Ethnic variation in LAI utilisation was marked, ranging from 12.9% in Nepalese patients to 48.7% among Syrians. Middle Eastern populations showed consistently higher LAI rates (Emirati 47.9%, Egyptian 47.6%, Jordanian 41.0%) compared to South Asian populations (Indian 18.0%, Bangladeshi 18.0%, Pakistani 26.3%). These differences persisted after adjustment for clinical factors.

### 3.7 Clozapine utilisation

Among 2,028 patients meeting TRS criteria (≥2 adequate trials of ≥28 days at therapeutic dose of distinct non-clozapine antipsychotics), 158 (7.8%) were initiated on clozapine. Of these, 21 (18.9%) were initiated on clozapine before formal TRS eligibility (defined as the end date of the second failed adequate trial). Among the 90 delayed initiators, median delay from TRS eligibility to clozapine initiation was 10.2 months (IQR 2.8–30.8), with 26.7% achieving initiation within 3 months. Patients tried a median of 3 antipsychotics (IQR 2–4) before clozapine, and 91.6% of clozapine recipients also received concurrent antipsychotic polypharmacy. Of the 158 clozapine initiators, 21 initiated before formal TRS eligibility, 90 initiated after a delay from eligibility, and 47 initiated at or near the time of eligibility attainment and were not classified as delayed.

Multivariable logistic regression identified independent predictors of clozapine receipt (N = 5,125; Pseudo *R* ^2^ = 0.17; Figure 6B). The strongest predictor was antipsychotic polypharmacy (OR 7.74, 95% CI 5.27–11.37, p < 0.001), followed by schizophrenia diagnosis (OR 5.43, 95% CI 3.45–8.53, p < 0.001) and LAI receipt (OR 1.92, 95% CI 1.45–2.54, p < 0.001). Age was modestly associated (OR 1.03 per year, 95% CI 1.02–1.05, p < 0.001). First-line FGA use was negatively associated (OR 0.58, 95% CI 0.43–0.77, p < 0.001). TRS status showed a negative association (OR 0.42, 95% CI 0.30–0.59, p < 0.001), reflecting that clozapine is the defining treatment for TRS and its receipt is inversely related to the TRS binary classification. Sex, nationality, and treatment era were not significant predictors.

### 3.8 Antipsychotic polypharmacy

Oral antipsychotic polypharmacy (two or more concurrent oral antipsychotics at any point during follow-up) was observed in 2,198 patients (42.9%). To enable comparison with international cross-sectional data, point-prevalence assessment was performed at fixed timepoints, yielding concurrent prescribing rates of 20.1% at 3 months, 21.8% at 6 months, and 20.4% at 12 months from treatment initiation (Table 6). The most frequent concurrent oral combinations were olanzapine plus risperidone (n = 955; 18.9%), haloperidol plus risperidone (n = 823; 16.2%), and aripiprazole plus risperidone (n = 741; 14.6%).

**TABLE 6:** Antipsychotic polypharmacy.

Metric	Value
Longitudinal polypharmacy	
All-routes polypharmacy, n (%)	4,079 (79.6%)
Oral polypharmacy, n (%)	2,198 (42.9%)
Cross-sectional point prevalence	
At 3 months	20.1%
At 6 months	21.8%
At 12 months	20.4%
Polypharmacy by TRS status	
TRS patients, n/N (%)	1,744/2,028 (86.0%)
Non-TRS patients, n/N (%)	454/3,097 (14.7%)
International comparator	
Global point prevalence Højlund et al. (2024)	33.2%

Longitudinal polypharmacy = receipt of two or more distinct antipsychotics prescribed within a 30-day window at any point during follow-up. All-routes includes intramuscular formulations co-prescribed with oral antipsychotics during inpatient episodes. Cross-sectional point prevalence assessed at fixed timepoints from treatment initiation among patients with active prescriptions at each timepoint. TRS, treatment-resistant schizophrenia.

### 3.9 Sensitivity analyses

Multiple sensitivity analyses were conducted to assess robustness of primary findings (Table 7). Varying the gap definition for treatment discontinuation (30, 60, and 90 days without a prescription) yielded median oral persistence of 32 days (IQR 4–60), 36 days (IQR 6–86), and 38 days (IQR 7–109), respectively, with Kaplan-Meier medians of 49, 82, and 120 days. Twelve-month Kaplan-Meier persistence rates were 8.2%, 23.0%, and 37.2% under the 30-, 60-, and 90-day gap definitions (Table 7). The persistence pattern remained consistent across all definitions, with the majority of discontinuations occurring within the first 60 days of treatment.

**TABLE 7:** Sensitivity analyses for treatment persistence outcomes.

Gap definition (days)	N	FC median (days)	IQR	Mean (days)	SD	KM median (days)	FC 30d	KM 30d	FC 90d	KM 90d	FC 180d	KM 180d	FC 365d	KM 365d
30	5,066	32	4–60	57.5	104.7	49	56.5%	57.0%	16.6%	35.5%	6.1%	19.3%	2.1%	8.2%
60	5,066	36	6–86	96.9	196.8	82	60.4%	61.9%	24.0%	47.3%	12.9%	35.1%	6.7%	23.0%
90	5,066	38	7–109	169.6	403.8	120	62.4%	64.3%	28.2%	53.5%	17.9%	45.1%	11.6%	37.2%

Persistence re-calculated using alternative gap definitions (30, 60, and 90 days) to assess robustness of primary findings. The gap definition specifies the number of consecutive days without an antipsychotic prescription required to classify discontinuation. In this table, FC, percentages represent the proportion of all patients (N = 5,066) remaining on treatment at each timepoint, regardless of follow-up duration (differs from Table 3, which restricts to patients with sufficient follow-up). KM, estimates are censoring-adjusted. Consistent patterns of poor early persistence were observed across all gap definitions. Abbreviations: IQR, interquartile range; SD, standard deviation; FC, fraction continuous; KM, Kaplan-Meier.

Restriction by diagnostic subgroup confirmed consistent findings: patients with schizophrenia only (F20; n = 1,935) showed a median persistence of 41 days, and excluding all F23-only patients (n = 3,300) yielded a median of 40 days (Supplementary Table S2). Restricting the cohort to patients with 12 or more months of follow-up (n = 2,061) yielded a higher median persistence of 62 days (Supplementary Table S3). Comparison of new-episode patients (n = 4,823) versus prevalent cases (n = 302) showed no significant difference in persistence (p = 0.659; Supplementary Table S4). Excluding patients from the early era (2018–2019; n = 4,181 remaining) produced similar results (median 34 days; Supplementary Table S5).

Across established-patient sensitivity subsets, 12-month Kaplan–Meier persistence remained stable at 23%–25%, supporting the robustness of the primary persistence findings (Supplementary Table S6). In a separate sensitivity analysis using a stricter treatment-resistant schizophrenia criterion of three or more adequate trials rather than two, 891 patients (17.4%) met treatment-resistant schizophrenia criteria. This subgroup showed higher clozapine utilisation (11.4% vs 7.8%), higher long-acting injectable use (68.9% vs 55.3%), and near-universal polypharmacy (95.7%), consistent with greater treatment complexity in more refractory patients (Supplementary Table S7).

Propensity-score weighted analyses comparing oral first-line FGA versus SGA treatment (all standardised mean differences <0.1) confirmed a persistence disadvantage for FGAs (HR 1.43, 95% CI 1.21–1.70, p < 0.001), with weighted Kaplan-Meier median persistence of 29 days for FGA *versus* 90 days for SGA. However, a sensitivity analysis excluding oral haloperidol (leaving only 35 non-haloperidol FGA patients) rendered the class difference non-significant (HR 0.56, 95% CI 0.32–0.96, p = 0.034; FGA median 150 days vs SGA 90 days), suggesting that the FGA persistence disadvantage was largely attributable to haloperidol used as a short-term bridging agent rather than a true antipsychotic class effect. This non-haloperidol FGA comparison should be interpreted as exploratory, given the small sample size (n = 35) and failure to achieve covariate balance (Supplementary Table S8).

## 4 Discussion

This study presents the largest and most comprehensive analysis of antipsychotic prescribing patterns in the Gulf region, encompassing 5,125 patients with schizophrenia spectrum disorders across 120+ nationalities treated at Al Amal Psychiatric Hospital, Dubai, between 2018 and 2025. The findings reveal several noteworthy patterns: a dual prescribing framework characterised by high first-generation antipsychotic (FGA) use for acute management but second-generation antipsychotic (SGA) dominance in planned maintenance therapy; remarkably poor treatment persistence; high rates of treatment modification and antipsychotic polypharmacy; relatively high long-acting injectable(s) (LAI) utilisation driven by treatment-resistant cases; substantial clozapine underutilisation; and significant ethnic variation in prescribing patterns across one of the most diverse patient populations ever studied in psychopharmacological research.

### 4.1 First-line antipsychotic selection

The observation that FGAs comprised 43.8% of all first-line prescriptions (predominantly haloperidol at 43.0%) initially appears discordant with contemporary guidelines recommending SGAs as first-line treatment (Keepers et al., 2020). However, the secondary analysis restricted to first oral antipsychotic prescriptions provides crucial context: when intramuscular formulations were excluded, SGAs accounted for 90.4% of first-line oral prescriptions, with olanzapine (43.5%), risperidone (25.3%), and aripiprazole (12.4%) comprising the dominant agents. This dual prescribing pattern, whereby haloperidol is administered intramuscularly for acute agitation before rapid transition to an oral SGA for maintenance therapy, is characteristic of acute inpatient psychiatric settings and has been documented in emergency psychiatric practice globally (Garriga et al., 2016).

The oral first-line prescribing profile closely mirrors international patterns. The olanzapine dominance (43.5%) is remarkably consistent with UK primary care data, where Hayes et al. (2024) reported olanzapine accounting for 43.3% of prescriptions among 309,378 patients (Richards-Belle et al., 2024). This preference is consistent with the evidence base from landmark effectiveness trials: the CATIE trial demonstrated that olanzapine had the lowest discontinuation rate (64% at 18 months) among SGAs (Lieberman et al., 2005), while the EUFEST trial showed that olanzapine achieved the highest 1-year persistence (67%) compared with haloperidol (28%) in first-episode patients (Kahn et al., 2008). The CUtLASS trial similarly found that 74% of olanzapine-treated patients remained on medication at study end (Jones et al., 2006). Collectively, these trials support the pragmatic selection of olanzapine as a first-line agent based on its favourable persistence profile, despite metabolic concerns.

Regionally, our oral prescribing patterns align broadly with the RECONNECT-S study (Alkhadhari et al., 2015), the only prior multi-country investigation from the Gulf region (N = 1,057 across Egypt, Saudi Arabia, and Gulf States), which reported 93.9% SGA use at discharge, with risperidone (40.3%) and olanzapine (32.5%) as the leading agents. However, our higher olanzapine-to-risperidone ratio may reflect temporal shifts in prescriber preference and the growing evidence base for olanzapine efficacy (Huhn et al., 2019; Lieberman et al., 2005; Kahn et al., 2008). In Qatar, Ouanes et al. (2020) reported 56.2% SGA monotherapy with 28.3% FGA–SGA combinations (Ouanes et al., 2020), while in Oman, Al Za’abi et al. (2014) found atypical antipsychotics accounting for 68.8% of prescriptions, with olanzapine comprising 48.1% of atypical prescriptions (Al Za’abi et al,. 2014). Our findings contrast markedly with older Palestinian data, where Sweileh et al. (2013) documented 85.7% FGA predominance, likely reflecting medication availability constraints rather than prescriber preference (Sweileh et al., 2013). Across Asia, the REAP 2016 survey (Dong 2019; N = 3,537) reported 80.8% SGA use, with risperidone (31%–35%) and olanzapine (15%–31%) as the most prescribed agents, showing broad consistency with our oral-only findings (Dong et al., 2019).

### 4.2 Treatment persistence

Treatment persistence on the first oral antipsychotic was notably poor, with a raw median of 36 days (IQR 6–86), a Kaplan-Meier median of 82 days, and only 23.0% of patients remaining on their initial agent at 1 year. These figures are lower than those reported in major international studies. In the CATIE trial, median time to discontinuation was approximately 6 months, with overall 18-month discontinuation at 74% (Lieberman et al., 2005). The EUFEST trial reported 47% discontinuation at 1 year among first-episode patients, with olanzapine persistence at 67% (Kahn et al., 2008). Finnish registry data showed median treatment episode duration of 11.4 months (Rubio et al., 2021). The clinical significance of such poor persistence is underscored by meta-analytic evidence demonstrating 65% 1-year relapse rates following antipsychotic discontinuation *versus* 27% with continued maintenance treatment (Leucht et al., 2012).

Several factors may explain the lower persistence observed in our cohort. First, methodological differences in gap definitions substantially influence persistence estimates (Mahlich et al., 2021); our primary analysis used a 60-day gap definition (Mahlich et al., 2021; Yan et al., 2018), and sensitivity analyses confirmed that even with a 90-day gap (Mahlich et al., 2021; Rasmussen et al., 2022), the Kaplan–Meier median persistence reached only 120 days. Second, the inclusion of oral haloperidol (8.6% of first oral prescriptions) with its uniformly poor persistence (median 20 days, 8.2% at 12 months) reflects its intentional use as a short-term bridging agent rather than failed maintenance therapy (Patel et al., 2018). Third, the acute inpatient setting and diagnostic heterogeneity of our cohort (including 35.7% with brief psychotic disorder) likely contribute to shorter treatment courses compared with studies focusing exclusively on chronic schizophrenia or first-episode psychosis (Stephen and Lui, 2025; Tiihonen et al., 2011). Importantly, sensitivity analyses restricting to patients with schizophrenia only (F20; n = 1,935; median 41 days) or excluding all F23-only patients (n = 3,300; median 40 days) yielded comparable persistence estimates, confirming that the inclusion of brief psychotic disorder did not substantially bias the primary findings (Supplementary Table S2). Furthermore, 12-month Kaplan-Meier persistence remained stable at 23%–25% across all sensitivity subsets, including restriction to patients with two or more clinical encounters and exclusion of patients with one to two prescriptions (Supplementary Table S6), indicating that poor long-term persistence is a robust finding not driven by transient or single-encounter patients. The UAE’s transient expatriate population may contribute to early loss to follow-up for administrative rather than clinical reasons, but the consistency of findings across these subsets suggests that non-clinical attrition alone does not account for the observed pattern. Nevertheless, the steep early attrition curve, with approximately one-third of patients discontinuing within 30 days, warrants clinical attention regardless of the underlying reasons.

Among individual oral antipsychotics, the persistence hierarchy (risperidone 40 days > quetiapine 38 days > aripiprazole 36 days > olanzapine 35 days) differs from international data where olanzapine typically demonstrates superior persistence (Lieberman et al., 2005; Kahn et al., 2008). However, the short median olanzapine persistence partly reflects the large number of patients (n = 2,204) prescribed this agent, many receiving it immediately after haloperidol as part of planned acute-to-maintenance transitions (Garriga et al., 2016; Patel et al., 2018; National Instit ute for Health and Care Excellence NICE, 2015). Propensity-score weighted analyses confirmed a persistence disadvantage for oral FGA first-line treatment (HR 1.43, 95% CI 1.21–1.70), with weighted Kaplan-Meier median persistence of 29 days for FGA *versus* 90 days for SGA. Notably, when oral haloperidol was excluded from the comparison, the FGA class effect became non-significant, demonstrating that the observed persistence disadvantage is attributable entirely to haloperidol’s role as a short-term bridging agent rather than representing a true class difference in treatment durability, consistent with EUFEST data showing haloperidol had the highest 1-year discontinuation rate (72%) among all agents studied (Kahn et al., 2008).

### 4.3 Treatment modifications and antipsychotic polypharmacy

The high treatment modification rate (60.1%) and the median time to first modification (10 days, IQR 0–130) are distinctive features of our cohort. While the CATIE trial similarly demonstrated that approximately 70%–75% of patients required treatment changes across its phases (8), the modifications observed here reflect a fundamentally different clinical pattern: planned sequential prescribing, where initial agents (particularly haloperidol) serve as acute symptom management tools with immediate co-initiation or transition to maintenance SGAs. The predominant treatment pathway (FGA to SGA transition, accounting for 31.2% of modifications) supports this interpretation. Augmentation (52.0%) was substantially more common than switching (18.7%), indicating that clinicians frequently added a second antipsychotic to the initial agent before deciding on a definitive maintenance regimen.

Antipsychotic polypharmacy was common, with 79.6% of patients receiving two or more concurrent antipsychotics when considering all routes, declining to 42.9% for oral prescriptions only. The oral-only rate provides a more meaningful comparator, as it excludes transient acute-to-maintenance overlap.

The longitudinal oral polypharmacy rate was 42.9%, reflecting any period of concurrent oral antipsychotic prescribing across the full follow-up. For a like-for-like comparison with the global point-prevalence estimate of 33.2% reported by Højlund et al. (2024) (Højlund et al., 2024), cross-sectional point prevalence was calculated at fixed timepoints. The rates were 20.1% at 3 months, 21.8% at 6 months, and 20.4% at 12 months. These cross-sectional figures are notably lower than both the global estimate of 33.2% and regional comparators, including Qatar (44.1%) (Ouanes et al., 2020) and Palestine (50.4%–53.1%) (Sweileh et al., 2013). They are also well below the upper Asian range reported in Singapore (70.3%–75.8%) (Dong et al., 2019). This indicates that concurrent antipsychotic prescribing in this cohort appears more conservative than suggested by longitudinal measures alone.

The all-route combinations were dominated by intramuscular haloperidol co-prescribed with oral SGAs, whereas oral-only combinations revealed frequent SGA–SGA pairing, particularly olanzapine plus risperidone (18.9%) and haloperidol plus risperidone (16.2%), suggesting that maintenance polypharmacy reflects deliberate multi-agent strategies beyond transitional overlap (Hasan et al., 2012; National Institute for Health and Care Excellence NICE, 2014).

The polypharmacy rates warrant careful interpretation. Unlike cross-sectional point-prevalence studies, our longitudinal measure captures any instance of overlapping antipsychotic prescriptions across the entire follow-up period, which inflates the estimate relative to cross-sectional assessments. The distinction between all-route (79.6%) and oral-only (42.9%) rates further demonstrates that a substantial proportion of apparent polypharmacy reflects planned short-term overlap during acute-to-maintenance transitions.

Nevertheless, Højlund et al. (2024) found that antipsychotic polypharmacy was associated with increased risk of relapse (RR 1.42), psychiatric hospitalisation (RR 1.24), and all-cause mortality (RR 1.19), underscoring the importance of distinguishing planned short-term overlap from sustained polypharmacy, though the high polypharmacy rates in the TRS subgroup likely reflect clinical complexity rather than inappropriate prescribing (Højlund et al., 2024).

### 4.4 Long-acting injectable utilisation

The LAI utilisation rate of 34.1% is higher than most international comparators. Finnish and Swedish registry data report LAI use at 12.8% and 21.6%, respectively (Taip et al., 2021), while Asian REAP survey data show wide variation in LAI use, ranging from 0% in Vietnam to 44.9% in Malaysia (Tang et al., 2020). The relatively high rate in our cohort is largely attributable to the substantial TRS population: 55.3% of TRS patients received LAI compared with only 20.3% of non-TRS patients. This treatment-resistance-driven LAI prescribing pattern contrasts with recommendations advocating earlier consideration of LAIs, particularly where adherence is a concern (Correll et al., 2016).

Paliperidone palmitate was the dominant LAI (59.8% of all LAI prescriptions), followed by aripiprazole LAI (30.9%) and haloperidol decanoate (9.3%). The preference for paliperidone palmitate is consistent with its favourable evidence base; Tiihonen et al. (2017) demonstrated that LAI paliperidone had the lowest rehospitalisation risk (HR 0.51) among all antipsychotics in a Swedish cohort of 29,823 patients (Tiihonen et al., 2017). Broader evidence supports LAI superiority over oral formulations, with Kishimoto et al. (2013) reporting a hospitalisation risk ratio of 0.43 for LAIs *versus* oral antipsychotics in a mirror-image meta-analysis (N = 5,940), and Rubio et al. (2021) finding that LAI users were 67% less likely to interrupt treatment (aHR 0.33) (Rubio et al., 2021; Kishimoto et al., 2021).

LAI utilisation differed substantially by nationality within a single healthcare system, and these differences remained after adjustment for measured clinical factors. Because nationality is an imperfect proxy for multiple unmeasured determinants, these patterns should be interpreted cautiously and not ascribing causality. Prior work in Western settings has documented group differences in LAI prescribing (Aggarwal et al., 2012; Wang et al., 2023), sometimes discussed in relation to inequities and coercive pathways to care (Barnett et al., 2019). In the UAE’s multinational context, observed differences in LAI use may reflect variation in service pathways and transitions of care (e.g., inpatient initiation *versus* outpatient initiation, and whether timely outpatient injections are arranged after discharge), health-system and administrative factors (e.g., coverage/authorisation processes and service infrastructure for delivering injections), and communication/shared decision-making factors (e.g., how injectable options are discussed, addressed, and aligned with patient preferences, including language or interpretation needs). These mechanisms require dedicated study in the UAE setting (Carroll et al., 2024; Getzen et al., 2013; Kane and Rubio, 2023; Robinson et al., 2020; Romstadt and Wonson, 2018; Throneberry et al., 2025).

### 4.5 Clozapine utilisation and treatment-resistant schizophrenia

TRS prevalence in our cohort (39.6%; n = 2,028) aligns with meta-analytic estimates (Siskind et al., 2022; Diniz et al., 1999), yet clozapine was initiated in only 7.8% of TRS patients (n = 158), consistent with marked international variation and low uptake reported across Gulf settings (Bachmann et al., 2017; Ouanes et al., 2020; Al-Huseini et al., 2025; Wadoo et al., 2022). This proportion is substantially lower than reported utilisation in Finland (22.0% of schizophrenia patients) (Taipale et al., 2021), China (31.9%) (Xu et al., 2020), and the UK, where approximately one-third of eligible TRS patients receive clozapine (Whiskey et al., 2021). Qualitative evidence from the Arabian Gulf suggests that underuse may reflect system and prescriber barriers, including monitoring burden and limited experience (Ismail et al., 2019).

Among delayed initiators (n = 90), the median time from TRS eligibility to clozapine initiation was 10.2 months (IQR 2.8–30.8), and only 26.7% started within 3 months. Delays matter clinically: response appears to decline with prolonged deferral, and each additional prior antipsychotic exposure has been linked to reduced likelihood of clozapine response (Nielsen et al., 2012; Yoshimura et al., 2017). Although the median delay here is shorter than multi-year delays reported elsewhere, it still exceeds guideline expectations (Howes et al., 2012; Shah et al., 2018). A stricter TRS definition (≥3 adequate trials, 4-week threshold) identified a more severe subgroup (17.4%; n = 891) with higher clozapine use (11.4%) and near-universal polypharmacy (95.7%), consistent with increasing treatment complexity as refractoriness progresses.

### 4.6 Concomitant psychotropic medication use

The extensive use of concomitant psychotropic medications, particularly benzodiazepines (79.8%) and anticholinergics (66.7%), is notable. Benzodiazepine co-prescription substantially exceeded most international comparators including CATIE (18%–22%; Chakos 2006) (Chakos et al., 2006), European data (32%) (Toto et al., 2019), and Asian rates (27.8%) (Dong et al., 2019), though it was comparable to figures from some settings; for example, Japanese schizophrenia samples report benzodiazepine-derivative anxiolytic use around 49.7% (Shinfuku et al., 2012), and benzodiazepine/Z-hypnotic prescribing at discharge from acute psychiatric inpatient care has been reported at approximately 30% in Scotland (Johnson et al., 2018).

Our rate also considerably exceeded those reported in other Gulf settings (Qatar 12.5%; Palestine 6.8%). This disparity is likely driven in part by the acute inpatient treatment context of our cohort: the temporal trend data demonstrate that an increasing proportion of patients present in acute agitation necessitating rapid tranquillisation, and benzodiazepines remain a cornerstone of acute agitation management protocols alongside intramuscular antipsychotics (Keepers et al., 2020; Rasmussen et al., 2022). The longitudinal measurement approach, which captures any benzodiazepine prescription across the entire follow-up period, further inflates the rate relative to cross-sectional assessments that capture prescribing at a single timepoint. Differences in prescribing culture, formulary composition, and the relative availability of alternative anxiolytic strategies may also contribute to variation across Gulf settings.

Anticholinergic prescribing was notably higher among FGA-initiated patients (85.0% *versus* 52.4% for SGA-initiated patients), consistent with the well-established greater extrapyramidal side-effect liability of first-generation agents and the resulting common use of anticholinergics to prevent or treat antipsychotic-induced movement disorders (Desmarais et al., 2012; Peluso et al., 2012). Antidepressant use (32.5%) was consistent with CATIE data (31%–38%) (Chakos et al., 2006) and supported by meta-analytic evidence of efficacy for both depressive and negative symptoms in schizophrenia (Helfer 2016). Mood stabiliser use (26.8%) exceeded most international comparators (Europe 16.2%–20.4%; Asia 13.6%–13.7%; CATIE 15%–18%), potentially reflecting the diagnostic complexity of our cohort (Dong et al., 2019; Chakos et al., 2006; Lim et al., 2020; Sim et al., 2011).

### 4.7 Ethnic and cultural variation

The representation of 120 nationalities within a single institution provides an unprecedented opportunity to examine ethnic variation in antipsychotic prescribing within a unified healthcare system. Prior research has largely been limited to comparisons within Western settings, where African-Americans patients in the US receive more haloperidol, more depot formulations, and less clozapine compared with Caucasian patients (Kuno and Rothbard, 2002; Medina et al., 2024; Ventura et al., 2022). UK data similarly show that Black patients are more likely to receive depot antipsychotics and less likely to receive SGAs or clozapine, patterns attributed in part to higher rates of compulsory admission and adversarial pathways to care (Wang et al., 2023; Barnett et al., 2019).

Against that backdrop, we observed ethnic variation across multiple prescribing domains in our setting, but with patterns that do not mirror Western racial disparities. Olanzapine predominated among African and South Asian patients, whereas Emirati patients showed a more balanced distribution with higher aripiprazole and quetiapine use. Long-acting injectable utilisation was highest among Middle Eastern populations and lowest among South Asian populations. Importantly, “ethnicity” in this context likely proxies a different and more complex set of determinants than in Western race-based analyses, including language, migration history, cultural models of mental illness, health literacy, insurance coverage, and access to follow-up care. Consistent with this interpretation, UAE nationality was associated with higher treatment modification rates (HR 1.23, 95% CI 1.12–1.34), which may reflect more aggressive treatment optimization enabled by the national insurance system and greater continuity of care.

### 4.8 Temporal trends

The temporal analysis revealed two striking trends: a progressive decline in SGA first-line prescribing from 86.0% in 2018 to 40.4% in 2025, driven by a six-fold increase in haloperidol use (14.0%–59.6%); and a 19-fold acceleration in time to LAI initiation for patients treated in 2024–2025 compared with 2018–2020 (HR 19.14, 95% CI 15.46–23.69). These trends move in opposite directions to international prescribing patterns. Globally, SGA use has been increasing: Asian data show SGA prescribing rising from 45.5% in 2001 to 73.7% in 2009 (Xiang et al., 2015), Hong Kong data show oral SGA use increasing from 23.8% to 54.1% between 2006 and 2016 (Fang et al., 2025), and Japanese data show SGA increases from 28.9% in 2001 to 70.3% in 2021 (Yasui-Furukori et al., 2023).

The apparent counter-trend in our setting reflects increasing use of intramuscular haloperidol for acute agitation management (Supplementary Table S1; Supplementary Figure S1) and growing patient volume at the facility, rather than a deliberate move towards FGA-first prescribing; no changes in formulary availability or insurance reimbursement occurred during the study period. The increasing proportion of patients presenting in acute agitation necessitating intramuscular haloperidol for rapid tranquillisation would inflate the all-routes FGA rate without necessarily indicating a change in maintenance prescribing philosophy. This interpretation is supported by the observation that oral SGA prescribing remained above 90% through 2023, suggesting that the earlier all-routes FGA increase reflected acute IM prescribing rather than a shift in maintenance prescribing philosophy. The subsequent decline in oral SGA prescribing in 2024–2025 may reflect a genuine change in prescribing practice that warrants monitoring in future studies. The acceleration in LAI initiation, in contrast, represents a genuine and clinically desirable trend toward earlier adoption of long-acting formulations, consistent with evolving international guidelines and the growing evidence base supporting LAI use even in early-phase illness (Tiihonen et al., 2017; Winter-van Rossum et al., 2023).

### 4.9 Strengths and limitations

This study has several strengths. To our knowledge, it represents one of the largest single-centre, longitudinal investigations of antipsychotic prescribing in a tertiary psychiatric setting in the Gulf region, and it exceeds the sample sizes of prior regional hospital-based studies (for example, Qatar n = 537; Oman n = 535) (Ouanes et al., 2020; Al Za’abi et al,. 2014). Although the RECONNECT-S Beta study enrolled 1,057 patients across multiple Middle Eastern countries, only 244 participants were from the Gulf States (Kuwait and the United Arab Emirates combined), highlighting the relative scarcity of large Gulf-based cohorts (Alkhadhari et al., 2015). The inclusion of 120 nationalities within a single healthcare system enables analysis of prescribing variation with reduced between-system confounding. The 2018–2025 study period supports temporal trend analysis. EMR prescribing data enhance ascertainment of issued treatment, and the use of complementary analytical approaches (Kaplan–Meier analysis, Cox regression, and logistic regression) with sensitivity analyses strengthens robustness.

Several limitations should be acknowledged. As a single-centre tertiary cohort, the case-mix inherently captures a higher proportion of severe, acute, and treatment-refractory cases. The prescribing patterns observed, including the 39.6% TRS prevalence, extensive intramuscular haloperidol use, and high benzodiazepine co-prescribing, likely overestimate the acuity and complexity of the broader regional schizophrenia population, and findings may not generalise to community, primary care, or outpatient settings across the Gulf region. These results are most directly applicable to treated schizophrenia-spectrum patients within tertiary psychiatric hospitals. EMR prescribing records capture prescriptions issued rather than adherence, so persistence based on prescription gaps may misclassify true medication-taking. The operationalised treatment-resistant schizophrenia definition (two “adequate” trials inferred from prescriptions) may not fully align with clinical treatment resistance because dosing adequacy, adherence, and symptomatic response could not be confirmed. As with all observational prescribing studies, confounding by indication remains a major limitation: medication choice, switching, LAI use, and clozapine initiation are influenced by symptom severity, agitation, adherence concerns, clinician preference, and service-level factors that were not fully captured in structured electronic medical records.

Clinical outcomes (symptom severity, functioning, and readmissions outside the institution) were unavailable. Finally, although variation by nationality/ethnic background was observed, we could not determine whether differences reflect appropriate individualisation or inequitable access because socioeconomic, linguistic, and insurance variables were incomplete; clozapine delays and polypharmacy are examined in greater depth in a companion analysis. Cumulative outcomes such as LAI receipt, clozapine initiation, and number of distinct antipsychotics are inherently follow-up-dependent, and patients with longer observation periods had more opportunity to accumulate these events; although sensitivity analyses restricted by follow-up duration yielded consistent findings, residual time-at-risk differences may still influence subgroup comparisons. Data extraction was performed on 22 December 2025. Sensitivity analyses excluding patients entering care after October 2025 confirmed that late entries with limited follow-up did not bias persistence estimates (KM median 83 *versus* 82 days for the full cohort) (Supplementary Table S9).

### 4.10 Clinical implications

These findings highlight several areas warranting clinical attention. The poor first-line persistence, with a raw median duration of 36 days and only 23.0% remaining at 1 year (Kaplan-Meier estimate), warrants systematic intervention including structured early review, shared decision-making, and psychoeducation about maintenance treatment. The substantial clozapine underutilisation (7.8% of TRS patients, with median delays of 10.2 months) represents a specific target for implementing standardised TRS identification and clozapine initiation pathways with prescriber education and monitoring support. The accelerated LAI adoption in recent cohorts is a positive trend that should be sustained and expanded beyond treatment-resistant populations. Finally, the ethnic variation in prescribing across 120 nationalities should prompt systematic evaluation to ensure equitable access to evidence-based treatments, including clozapine and LAI formulations, across all patient groups.

## 5 Conclusion

This study provides the most detailed characterisation of antipsychotic prescribing patterns in the Gulf region to date. While oral first-line prescribing broadly aligns with international SGA-first guidelines, the findings highlight several areas requiring attention: poor treatment persistence, clozapine underutilisation, and longitudinal polypharmacy rates that appear high but cross-sectional point prevalence that falls below international benchmarks, and ethnic variation in prescribing across one of the world’s most diverse patient populations. The accelerating adoption of LAI formulations represents a positive development, but systematic efforts to improve clozapine access, evaluate polypharmacy patterns in the context of clinical complexity, and ensure equitable prescribing across ethnic groups are needed to optimise outcomes for patients with schizophrenia spectrum disorders in the UAE and similar multinational healthcare settings.

## Data Availability

The original contributions presented in the study are included in the article/Supplementary Material, further inquiries can be directed to the corresponding author.
