# Supplementary material for: Real-world antipsychotic prescribing pathways and treatment modifications in schizophrenia spectrum disorders: evidence from the United Arab Emirates
Source: Front Pharmacol. 2026 May 29;17:1808636. doi: 10.3389/fphar.2026.1808636 (PMC13260491; doi:10.3389/fphar.2026.1808636)
Supplement: Supplementary file 1 [file DataSheet2.docx]

**Supplementary Appendix**

**STROBE Statement—Checklist of items that should be included in reports of *cohort studies***

|  | Item No | Recommendation | Page No |
| --- | --- | --- | --- |
| **Title and abstract** | 1 | (*a*) Indicate the study’s design with a commonly used term in the title or the abstract | “Abstract”; “2.1 Study Design and Setting”. |
|  |  | (*b*) Provide in the abstract an informative and balanced summary of what was done and what was found | “Abstract” (Background, Methods, Results, Conclusion). |
| Introduction | | | |
| Background/rationale | 2 | Explain the scientific background and rationale for the investigation being reported | “1 Introduction”. |
| Objectives | 3 | State specific objectives, including any prespecified hypotheses | “1 Introduction”. |
| Methods | | | |
| Study design | 4 | Present key elements of study design early in the paper | “Abstract”; “2.1 Study Design and Setting”. |
| Setting | 5 | Describe the setting, locations, and relevant dates, including periods of recruitment, exposure, follow-up, and data collection | “2.1 Study Design and Setting” (1 January 2018 to 22 December 2025). |
| Participants | 6 | (*a*) Give the eligibility criteria, and the sources and methods of selection of participants. Describe methods of follow-up | “2.2 Study Population”; “3.1 Study Population”; Figure 1). |
|  |  | (*b*) For matched studies, give matching criteria and number of exposed and unexposed | Not applicable (unmatched retrospective cohort study). |
| Variables | 7 | Clearly define all outcomes, exposures, predictors, potential confounders, and effect modifiers. Give diagnostic criteria, if applicable | “2.4 Outcomes and Operational Definitions”; “2.5 Treatment-Resistant Schizophrenia Definition”. |
| Data sources/ measurement | 8* | For each variable of interest, give sources of data and details of methods of assessment (measurement). Describe comparability of assessment methods if there is more than one group | “2.3 Data Sources”; “2.4 Outcomes and Operational Definitions”. |
| Bias | 9 | Describe any efforts to address potential sources of bias | “2.3 Data Sources” (blinded extraction, programmatic standardisation, pre-specified analysis plan); “2.7 Sensitivity Analyses”. |
| Study size | 10 | Explain how the study size was arrived at | “2.2 Study Population”; “3.1 Study Population”; Figure 1. No a priori sample-size calculation; all eligible patients during the study period were included. |
| Quantitative variables | 11 | Explain how quantitative variables were handled in the analyses. If applicable, describe which groupings were chosen and why | “2.4 Outcomes and Operational Definitions” (persistence, modifications, LAI, polypharmacy); “2.5 Treatment-Resistant Schizophrenia Definition” (dose and duration thresholds); “2.6 Statistical Analysis”. |
| Statistical methods | 12 | (*a*) Describe all statistical methods, including those used to control for confounding | “2.6 Statistical Analysis” (Kaplan–Meier, Cox proportional hazards, logistic regression, inverse-probability-of-treatment weighting with propensity scores including age, sex, nationality, diagnosis, era, and comorbidities). |
|  |  | (*b*) Describe any methods used to examine subgroups and interactions | “2.6 Statistical Analysis” (propensity-score weighted class comparison); “2.7 Sensitivity Analyses” (diagnostic subgroups, TRS subgroups, era subgroups). |
|  |  | (*c*) Explain how missing data were addressed | “2.2 Study Population” (missing data minimal: age/sex complete; nationality <1% missing, coded as “Unknown”); “2.6 Statistical Analysis” (complete-case analysis for all multivariable models). |
|  |  | (*d*) If applicable, explain how loss to follow-up was addressed | “2.4 Outcomes and Operational Definitions” (Kaplan–Meier censoring-adjusted persistence to account for incomplete follow-up); “2.6 Statistical Analysis”; “2.7 Sensitivity Analyses” (restriction to ≥12 months follow-up; late-entry exclusions). |
|  |  | (*e*) Describe any sensitivity analyses | “2.7 Sensitivity Analyses”. |
| Results | | |  |
| Participants | 13* | (a) Report numbers of individuals at each stage of study—eg numbers potentially eligible, examined for eligibility, confirmed eligible, included in the study, completing follow-up, and analysed | “3.1 Study Population”; Figure 1: 22,578 unique patients screened; 5,285 with F20–F29 diagnoses; 160 excluded (no antipsychotic prescription); final analytic cohort N=5,125 (oral first-line subcohort N=5,066). |
|  |  | (b) Give reasons for non-participation at each stage | “3.1 Study Population”; Figure 1. |
|  |  | (c) Consider use of a flow diagram | Figure 1. |
| Descriptive data | 14* | (a) Give characteristics of study participants (eg demographic, clinical, social) and information on exposures and potential confounders | “3.1 Study Population”; Table 1: demographics, nationality, era, diagnosis, TRS status, comorbid SUD, and concomitant psychotropic use. |
|  |  | (b) Indicate number of participants with missing data for each variable of interest | “2.2 Study Population” (age and sex complete for all; nationality missing for <1% and coded as “Unknown”). |
|  |  | (c) Summarise follow-up time (eg, average and total amount) | “2.2 Study Population” (median follow-up 2.3 years, IQR 0.8–4.7; era distribution: 34.2% in 2018–2020, 46.5% in 2021–2023, 19.3% in 2024–2025). |
| Outcome data | 15* | Report numbers of outcome events or summary measures over time | “3.2 First-Line Antipsychotic Selection” to “3.8 Antipsychotic Polypharmacy”; Tables 2–6; Figures 2–5. |
| Main results | 16 | (a) Give unadjusted estimates and, if applicable, confounder-adjusted estimates and their precision (eg, 95% confidence interval). Make clear which confounders were adjusted for and why they were included | “3.4 Treatment Persistence” (Kaplan–Meier estimates with CIs); “3.5 Treatment Modifications” (Cox HRs with 95% CIs); “3.6 Long-Acting Injectable Utilisation” (logistic ORs with 95% CIs); “3.7 Clozapine Utilisation” (logistic ORs with 95% CIs); “3.9 Sensitivity Analyses” (propensity-score weighted HR); Tables 3, 7; Figure 5. Confounders listed in “2.6 Statistical Analysis”. |
|  |  | (b) Report category boundaries when continuous variables were categorized | “2.5 Treatment-Resistant Schizophrenia Definition” (adequate trial: ≥28 days [4-week] at therapeutic dose, defined as midpoint of licensed range or chlorpromazine equivalent ≥400 mg/day; stricter sensitivity definition: ≥42 days [6-week] and ≥3 trials); “2.7 Sensitivity Analyses” (30-, 60-, and 90-day gap definitions for discontinuation). |
|  |  | (c) If relevant, consider translating estimates of relative risk into absolute risk for a meaningful time period | “3.4 Treatment Persistence” (absolute 12-month Kaplan–Meier persistence 23.0%, 6-month 35.1%); Table 3; Table 7 for absolute persistence rates by gap definition. |
| Other analyses | 17 | Report other analyses done—eg analyses of subgroups and interactions, and sensitivity analyses | “3.9 Sensitivity Analyses”; Table 7; Supplementary Tables S1–S9 and Supplementary Figure S1. Pre-specified subgroup and sensitivity analyses by gap definition, follow-up duration, diagnostic subset, prescription/encounter count, TRS criteria, era, and haloperidol exclusion. |
| Discussion | | | |
| Key results | 18 | Summarise key results with reference to study objectives | “4 Discussion” (opening); “5 Conclusion”. |
| Limitations | 19 | Discuss limitations of the study, taking into account sources of potential bias or imprecision. Discuss both direction and magnitude of any potential bias | “4.9 Strengths and Limitations”. |
| Interpretation | 20 | Give a cautious overall interpretation of results considering objectives, limitations, multiplicity of analyses, results from similar studies, and other relevant evidence | “4.1 First-Line Antipsychotic Selection” to “4.10 Clinical Implications”; “5 Conclusion”. |
| Generalisability | 21 | Discuss the generalisability (external validity) of the study results | “2.1 Study Design and Setting” (catchment paragraph); “4.9 Strengths and Limitations”. |
| Other information | | | |
| Funding | 22 | Give the source of funding and the role of the funders for the present study and, if applicable, for the original study on which the present article is based | “Funding” (no financial support received for the research, authorship, or publication of this article). |

*Give information separately for exposed and unexposed groups.

**Note:** An Explanation and Elaboration article discusses each checklist item and gives methodological background and published examples of transparent reporting. The STROBE checklist is best used in conjunction with this article (freely available on the Web sites of PLoS Medicine at http://www.plosmedicine.org/, Annals of Internal Medicine at http://www.annals.org/, and Epidemiology at http://www.epidem.com/). Information on the STROBE Initiative is available at http://www.strobe-statement.org.
