# Supplementary material for: Real-world antipsychotic prescribing pathways and treatment modifications in schizophrenia spectrum disorders: evidence from the United Arab Emirates
Source: Front Pharmacol. 2026 May 29;17:1808636. doi: 10.3389/fphar.2026.1808636 (PMC13260491; doi:10.3389/fphar.2026.1808636)
Supplement: Supplementary file 2 [file DataSheet1.docx]

**Supplementary Material**

**Supplementary Table S1. First-Line Haloperidol Prescriptions by Route of Administration and Year**

| **Year** | **Total 1st** | **IM N** | **IM %** | **Oral N** | **Oral %** |
| --- | --- | --- | --- | --- | --- |
| 2018 | 584 | 39 | 6.7% | 15 | 2.6% |
| 2019 | 330 | 92 | 27.9% | 10 | 3.0% |
| 2020 | 840 | 300 | 35.7% | 9 | 1.1% |
| 2021 | 804 | 305 | 37.9% | 22 | 2.7% |
| 2022 | 818 | 378 | 46.2% | 39 | 4.8% |
| 2023 | 760 | 350 | 46.1% | 46 | 6.1% |
| 2024 | 707 | 338 | 47.8% | 94 | 13.3% |
| 2025 | 282 | 17 | 6.0% | 151 | 53.5% |

**Note.** No haloperidol decanoate (LAI) as first-line in any year. No drug shortages or insurance reimbursement changes occurred during the study period.

**Supplementary Table S2. Diagnostic Stratification of Brief Psychotic Disorder (F23) Patients**

| **Category** | **N** | **%** | **Median** | **IQR** | **KM Median** | **KM 12mo** |
| --- | --- | --- | --- | --- | --- | --- |
| Total with F23 in history | 2101 | 41.0% | 34 | 4–48 | 93 | 17.5% |
| F23 with F20/F25 also in history | 276 | 13.1% of F23 | 36 | 6–98 | 60 | 14.0% |
| F23-only (no F20/F25) | 1825 | 86.9% of F23 | 34 | 4–46 | 111 | 19.0% |
| **F23-only stratification** |  |  |  |  |  |  |
| Chronic engagement (>6 months) | 253 | 13.9% | 41 | 15–122 | 51 | 14.8% |
| Intermediate (1–6 months) | 217 | 11.9% | 53 | 11–90 | N/R | N/R |
| Brief (all within 1 month) | 1355 | 74.2% | 33 | 3–40 | 111 | N/R |
| **Comparator** |  |  |  |  |  |  |
| F20 schizophrenia | 1935 | 37.8% | 41 | 6–130 | 68 | 23.0% |
| Excluding all F23-only | 3300 | 64.4% | 40 | 6–121 | 68 | 22.7% |

**Note.** Patients with F23 codes stratified by clinical engagement pattern. Persistence on first oral antipsychotic was consistent across subgroups. N/R = not reached (median not estimable due to high censoring). IQR = interquartile range of raw persistence in days.

**Supplementary Table S3. Restriction to Patients with ≥12 Months Follow-up**

| **Cohort** | **N** | **Median** | **LAI %** | **CLZ %** | **Poly %** | **Mean APs** | **% Change** |
| --- | --- | --- | --- | --- | --- | --- | --- |
| Full Oral Cohort | 5066 | 36 | 34.1% | 5.1% | 42.9% | 3.0 | 60.1% |
| ≥12 Months Follow-up | 2061 | 62 | 55.7% | 9.1% | 67.2% | 4.0 | 85.2% |

**Supplementary Table S4. New Episode versus Prevalent Patients**

| **Cohort** | **N** | **Median** | **LAI %** | **CLZ %** | **Poly %** | **Mean APs** | **% Change** |
| --- | --- | --- | --- | --- | --- | --- | --- |
| New Episode | 4823 | 36 | 33.4% | 5.0% | 42.8% | 3.0 | 59.4% |
| Prevalent | 302 | 42 | 45.7% | 6.6% | 44.4% | 3.0 | 59.9% |

**Supplementary Table S5. Excluding Early Era (2018–2019)**

| **Cohort** | **N** | **Median** | **LAI %** | **CLZ %** | **Poly %** | **FGA 1st %** | **SGA 1st %** |
| --- | --- | --- | --- | --- | --- | --- | --- |
| Full Cohort | 5066 | 36 | 34.1% | 5.1% | 42.9% | 9.6% | 90.4% |
| Excluding 2018–2019 | 4181 | 34 | 31.6% | 4.6% | 39.9% | 10.3% | 89.7% |

**Supplementary Table S6. Persistence Sensitivity: Established Patient Status (N=5,125)**

| **Subset** | **N** | **Median** | **IQR** | **KM Median** | **KM 12mo** |
| --- | --- | --- | --- | --- | --- |
| All AP-exposed patients | 5125 | 36 | 6–86 | 82 | 23.0% |
| Excluding 1–2 Rx | 4322 | 40 | 11–98 | 101 | 25.3% |
| ≥2 encounters | 3656 | 43 | 11–120 | 93 | 25.2% |
| ≥3 encounters | 2965 | 51 | 13–153 | 92 | 25.1% |

**Note.** 12-month KM persistence stable at 23–25% across all subsets. IQR = interquartile range of raw persistence in days. Analysis includes all patients with antipsychotic exposure (N=5,125); the oral-specific cohort (N=5,066) yields comparable results.

**Supplementary Table S7. Treatment-Resistant Schizophrenia Sensitivity Definitions**

| **Definition** | **N** | **% Cohort** | **CLZ N** | **CLZ %** | **LAI N** | **LAI %** | **Poly %** | **Mean APs** | **Med Pers** |
| --- | --- | --- | --- | --- | --- | --- | --- | --- | --- |
| ≥2 trials (4-week) | 2028 | 39.6% | 158 | 7.8% | 1121 | 55.3% | 86.0% | 4.3 | 56 |
| ≥2 trials (6-week) | 1617 | 31.6% | 146 | 9.0% | 944 | 58.4% | 88.1% | 4.5 | 72 |
| ≥3 trials (4-week) | 891 | 17.4% | 102 | 11.4% | 614 | 68.9% | 95.7% | 5.4 | 62 |
| ≥3 trials (6-week) | 694 | 13.5% | 95 | 13.7% | 491 | 70.7% | 97.4% | 5.6 | 78 |

**Note.** Adequate trial: 4-week = ≥28 days; 6-week = ≥42 days at therapeutic dose. CLZ = Clozapine. LAI = long-acting injection. Poly = polypharmacy. Mean APs = mean number of distinct antipsychotics. Med Pers = raw median persistence in days.

**Supplementary Table S8. Propensity-Score Weighted FGA vs SGA Comparison**

| **Analysis** | **N FGA** | **N SGA** | **Max SMD** | **Weighted HR (95% CI)** | **KM medians** |
| --- | --- | --- | --- | --- | --- |
| With nationality | 485 | 4581 | 0.09 | 1.43 (1.21–1.70), p<0.001 | FGA 29d vs SGA 90d |
| Excluding haloperidol | 35 | 4581 | 0.83* | 0.56 (0.32–0.96), p=0.034 | FGA 150d vs SGA 90d |

**Note.** *Balance not achieved with only 35 non-haloperidol FGA patients. Covariates: age, sex, nationality, diagnosis, era, comorbidities.

**Supplementary Table S9. Late-2025 Exclusion Sensitivity**

| **Subset** | **N** | **Median** | **KM Median** | **KM 12mo** |
| --- | --- | --- | --- | --- |
| All patients | 5125 | 36 | 82 | 23.0% |
| Before Oct 2025 | 5077 | 36 | 83 | 23.2% |
| Before Jul 2025 | 5004 | 36 | 84 | 23.2% |
| Before 2024 | 4136 | 37 | 86 | 24.3% |

**Note.** Sensitivity analyses excluding patients entering care in late 2025, demonstrating that short follow-up periods for recent entries did not bias persistence estimates.

**Supplementary Figure S1**. Haloperidol First-Line Prescribing by Route of Administration (2018–2025).


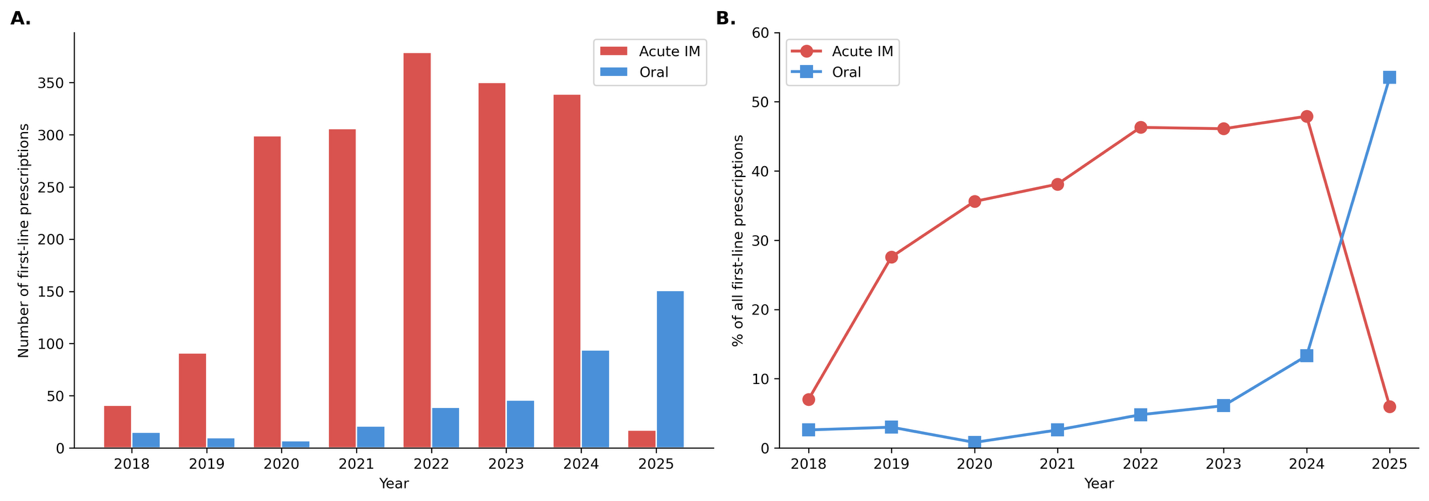


Note. **(A)** Absolute counts of first-line haloperidol prescriptions by route category (acute intramuscular versus oral). **(B)** Haloperidol prescriptions as a percentage of all first-line antipsychotic prescriptions by year. The increase in haloperidol use was driven predominantly by intramuscular formulations for acute agitation management between 2018 and 2024, whereas 2025 showed a shift toward oral haloperidol prescribing. No haloperidol decanoate (LAI) prescriptions were identified as first-line treatment in any year. Abbreviations: IM = intramuscular; LAI = long-acting injectable.
